# *In situ* analysis of gas dependent redistribution kinetics in bimetallic Au-Pd nanoparticles†

**DOI:** 10.1039/d4ta03030c

**Published:** 2024-06-27

**Authors:** Marta Perxés Perich, Christopher R. O'Connor, Koen M. Draijer, Nienke L. Visser, Nongnuch Artrith, Christian Reece, Petra E. de Jongh, Jessi E. S. van der Hoeven

**Affiliations:** a Materials Chemistry and Catalysis, Debye Institute for Nanomaterials Science, Utrecht University 3584 CG Utrecht The Netherlands j.e.s.vanderhoeven@uu.nl; b Rowland Institute at Harvard, Harvard University Cambridge Massachusetts 02142 USA

## Abstract

The catalytic and plasmonic properties of bimetallic gold–palladium (Au-Pd) nanoparticles (NPs) critically depend on the distribution of the Au and Pd atoms inside the nanoparticle bulk and at the surface. Under operating conditions, the atomic distribution is highly dynamic. Analyzing gas induced redistribution kinetics at operating temperatures is therefore key in designing and understanding the behavior of Au-Pd nanoparticles for applications in thermal and light-driven catalysis, but requires advanced *in situ* characterization strategies. In this work, we achieve the *in situ* analysis of the gas dependent alloying kinetics in bimetallic Au-Pd nanoparticles at elevated temperatures through a combination of CO-DRIFTS and gas-phase *in situ* transmission electron microscopy (TEM), providing direct insight in both the surface- and nanoparticle bulk redistribution dynamics. Specifically, we employ a well-defined model system consisting of colloidal Au-core Pd-shell NPs, monodisperse in size and uniform in composition, and quantify the alloying dynamics of these NPs in H_2_ and O_2_ under isothermal conditions. By extracting the alloying kinetics from *in situ* TEM measurements, we show that the alloying behavior in Au-Pd NPs can be described by a numerical diffusion model based on Fick's second law. Overall, our results indicate that exposure to reactive gasses strongly affects the surface composition and surface alloying kinetics, but has a smaller effect on the alloying dynamics of the nanoparticle bulk. Both our *in situ* methodology as well as the quantitative insights on restructuring phenomena can be extended to a wider range of bimetallic nanoparticle systems and are relevant in understanding the behavior of nanoparticle catalysts under operating conditions.

## Abstract 2

In this paper we present the *in situ* analysis of gas dependent alloying in bimetallic Au-Pd nanoparticles through a combination of CO-DRIFTS and *in situ* TEM providing direct insight in the surface- and nanoparticle bulk redistribution kinetics.

## Graphical abstract

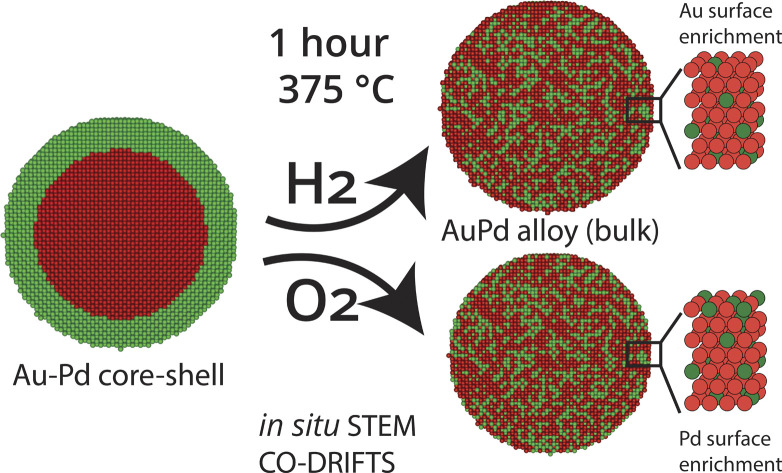

## Introduction

Bimetallic Au-Pd nanoparticles have extensively been explored in the fields of catalysis and plasmonics due to their favorable combination of optical and catalytic properties.^1–3^ Typically, Au offers high chemoselectivity in catalysis and a strong plasmonic response, whereas Pd provides high catalytic activity.^3–6^ Au-Pd NPs therefore present a valuable class of materials for thermal, photothermal and plasmon enhanced catalysis applications.^1,2,4,7^ In thermal catalysis, Au-Pd nanoparticles are valuable materials for selective hydrogenation and oxidation catalysis, where Pd facilitates fast H_2_ and O_2_ dissociation while Au steers the selectivity towards the desired partially hydrogenated or oxidated products.^3,7–10^ In both thermal and light enhanced catalytic applications, the metal distribution of the NP surface and NP bulk are critical. The surface composition and nature of the active sites directly affect the catalytic activity and selectivity of the Au-Pd NPs,^11–13^ whereas the metal distribution of the NP bulk also impacts the plasmonic response of the metal nanoparticles.^14–16^ For instance, only by combining Au and Pd in a core–shell structure, enhanced catalytic performances compared to Pd could be achieved, while retaining most of the favorable plasmonic properties of Au.^17^ Hence, achieving a fine control over the metal distribution is critical in tuning the plasmonic and catalytic properties of Au-Pd NP systems.

The metal distribution within bimetallic nanoparticles is dynamic and can change under reaction conditions.^18–21^ Metastable structures such as Au–Pd core–shell NPs can exhibit strongly enhanced catalytic activity and selectivity compared to their alloyed counterparts,^17^ but are prone to restructure under reactive gasses and at elevated temperatures. Therefore, it is relevant to gain a detailed understanding of gas-dependent restructuring. Thermodynamically, Au should dominate the surface of Au-Pd NPs, because Au has a lower surface energy than Pd under vacuum.^22^ Indeed, Au surface segregation has been observed when Au-Pd NPs were heated under vacuum or H_2_.^23–26^ However, under oxidizing conditions Pd can segregate to the surface through formation of an oxidized palladium layer, lowering the surface energy of the Pd atoms.^27,28^ Theoretical studies based on density functional theory (DFT) have indeed confirmed Pd stabilization on the surface in the presence of O_2_.^27,29^ However, most experimental and theoretical studies have focused on the final surface state after thermal treatment, whereas the dynamics of metal redistribution in reactive gasses remain unexplored for Au-Pd nanoparticle systems.

Direct observation of metal redistribution in bimetallic nanoparticles requires *in situ* techniques that can operate at high temperature and under gas atmospheres. Useful techniques include CO-DRIFTS, *in situ* EXAFS, and *in situ* TEM, all providing complementary information. CO adsorption measured by DRIFTS (diffuse reflectance infrared Fourier transform spectroscopy) is a surface specific, ensemble averaged technique that provides information both on the surface composition and nature of CO-adsorption sites averaged over a large number of Au-Pd NPs. Previous CO-DRIFTS studies on dilute Pd-in-Au nanoparticle alloys confirmed Au surface segregation upon annealing under vacuum^25^ and Pd segregation under O_2_ atmosphere,^30^ matching with theory.^27^ Additionally, CO-DRIFTS has been used to identify the Pd ensemble size at the nanoparticle surface, where the presence of Pd monomers, dimers or trimers in the NP surface can result in different catalytic properties.^31^ On the other hand, X-ray spectroscopy techniques such as EXAFS (extended X-ray absorption fine structure) have been used to analyze the metal distribution in dilute Pd-in-Au alloys after thermal treatment. The results revealed increased Pd–Pd bonds following O_2_ pretreatment, indicating Pd-segregation.^31^ Ensemble techniques such as CO-DRIFTS and EXAFS provide averaged information about the sample, but have difficulties in assessing differences on a single nanoparticle level.

*In situ* gas-phase transmission electron microscopy (TEM) allows direct visualization of changes in individual metal nanoparticles at elevated temperatures and/or upon exposure to reactive gasses.^32–34^ So far, several TEM studies on alloying dynamics of core–shell nanoparticles under high vacuum have been reported, using EDX (energy-dispersive X-ray) mapping,^35^ fast tomography,^36,37^ and atomic resolution imaging.^38,39^ However, these methods are difficult to utilize in the presence of a gas atmosphere because (1) EDX is time consuming and requires a high electron dose rate, which might lead to undesired beam effects, (2) the fast tomography approach is not possible with the current *in situ* TEM holder technologies, which do not allow tilting at the needed range, and (3) high resolution studies are often limited to a few particles only, neglecting effects caused by polydispersity within a sample. Assessing the effect of a gas atmosphere on the alloying dynamics of NPs has therefore remained unexplored and requires new methodologies to monitor and extract the alloying kinetics from *in situ* gas-phase TEM data.

In this work, we combine time-lapse CO-DRIFTS and *in situ* gas-phase TEM to directly observe the effect of a reducing and oxidizing gas atmosphere on the surface and bulk alloying kinetics of Au-Pd nanoparticles. Using these techniques, the alloying process of a well-defined model system consisting of colloidally prepared Au-core Pd-shell nanoparticles was assessed at the NP surface (CO-DRIFTS) and in the NP bulk (*in situ* TEM). Specifically, we focus on core–shell NPs with a 20 nm Au-core and a 1.7 nm-Pd shell, which are directly relevant for selective hydrogenation^17,40^ and oxidation^40,41^ catalysis. We developed a methodology to quantify the alloying dynamics of core–shell NPs from high angular annular dark field scanning transmission electron microscopy (HAADF-STEM) images acquired at atmospheric pressure without the need of EDX, fast tomography or atomic resolution imaging. Using this approach, we assess for the first time the NP bulk alloying dynamics from the *in situ* TEM data and show that the alloying dynamics can be predicted using a simple diffusion model based on Fick's second law.

## Results

### Preparation of the Au-core Pd-shell nanoparticles

**Fig. 1:**
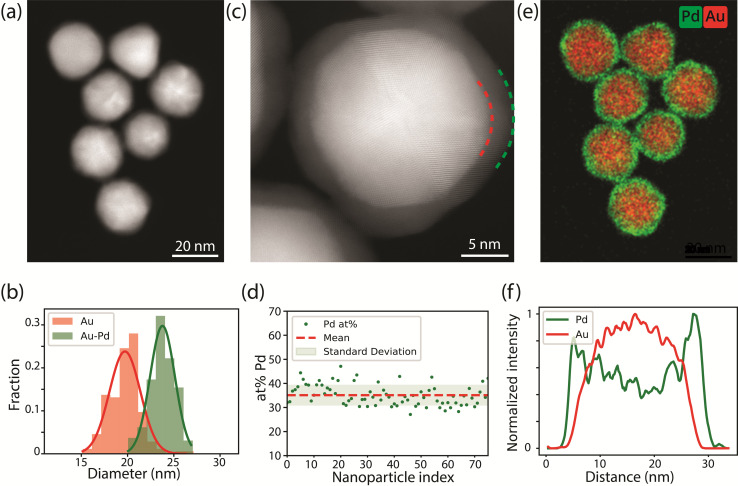
Electron microscopy characterization of the as-synthesized Au-Pd core–shell nanoparticles. (a) Representative HAADF-STEM image. (b) Size distribution of the Au (red) and Au–Pd (green) NPs, obtained by measuring more than 200 NPs. (c) High resolution HAADF-STEM image of the core–shell NPs, clearly showing the 1.7 nm Pd-shell surrounding the 19.7 nm Au-core, and the overall twinned symmetry of the Au-core Pd-shell particle. The red and green dashed lines highlight the interface between the Au-core and the Pd-shell and the end of the Pd-shell respectively. (d) Pd content (in atom percent, at%) of 75 individual NPs, derived from EDX analysis. (e) Corresponding EDX map of (a) showing Au and Pd in red and green, respectively. (f) Line scan of the NP in the bottom of the EDX map in frame (e).

Au-Pd core–shell nanoparticles (NPs) monodisperse in size and composition were successfully synthesized *via* colloidal synthesis. The high angular annular dark field scanning transmission electron microscopy (HAADF-STEM) image (Fig. 1a) and size distribution (Fig. 1b) show that the Au-core Pd-shell particles have an overall diameter of 23.8 ± 1.3 nm, consisting of a 19.7 ± 1.7 nm Au-core and a 1.7 ± 0.4 nm Pd-shell. The 1.7 nm Pd-shell is clearly visible in the high resolution HAADF-STEM image (Fig. 1c), which also reveals the epitaxial growth of Pd onto the Au-surface and the overall penta-twinned symmetry, as expected from the synthesis protocol.^5,13,42^ The presence of the Pd-shell and the uniformity of the metal composition among the different particles was further confirmed by EDX mapping (Fig. 1d and e). The corresponding line scan in Fig. 1f clearly demonstrates a core–shell structure, where the Pd signal extends further than the Au signal. EDX quantification also revealed that the average atomic percentage of palladium was 36 ± 5% among nearly all particles, which is close to the 31.5 atomic% Pd obtained from bulk analysis with inductive coupled plasma (ICP). Prior to all following TEM experiments, the polyvinylpyrrolidone ligands present on the surface of the NPs were removed using an activated carbon method.^43^

The Au-Pd core–shell NPs were supported on a silica support for the CO-DRIFTS measurements. TEM images show that the Au-Pd/SiO_2_ were homogeneously distributed over the support and that the core–shell structure was maintained after calcination at 300 °C for 3 h (Fig. S1†). This thermal treatment was sufficient to remove the PVP ligands present on the NPs' surface, as confirmed by thermogravimetric analysis (Fig. S2†). Note that PVP is more easily removed from the metal surface and that any remainders are likely to be on the silica support only.^44^ Thus, we expect that the Au-core Pd-shell NPs had a ligand free metal surface, both during CO-DRIFTS and TEM analysis.

### *Ex situ* TEM analysis of the alloying process

**Fig. 2:**
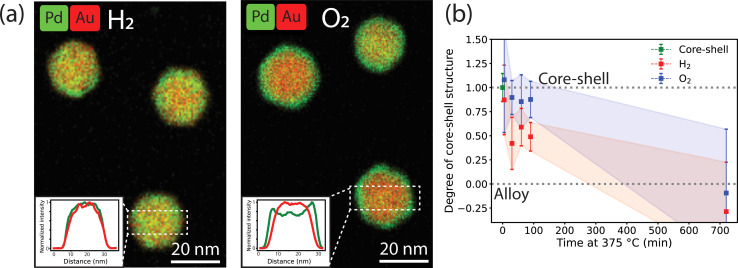
*Ex situ* EDX-STEM measurements of the alloying within the Au-core Pd-shell NPs in reducing and oxidizing gas atmospheres. (a) EDX maps after 60 min at 375 °C in 10% H_2_ in Ar and 10% O_2_ in Ar. The insets show line-scans of the selected rectangles. (b) Degree of core–shell structure calculated from the line scans of the EDX maps after heating at different times in a H_2_ or O_2_ atmosphere. The shadowed areas represent the standard deviation in the average from at least 8 line scans of 8 different nanoparticles per heating time. A value of 1.0 indicates a core–shell structure and a value of 0.0 a complete alloy. Examples of images and line scans at each time point are shown in Fig. S3.†

To study the effect of the gas atmosphere on the alloying process in Au-Pd NPs, *ex situ* analysis after treatment under isothermal conditions (375 °C) in H_2_ and O_2_ were conducted. The results in Fig. 2 show that depending on the gas atmosphere, the metal distribution of Au-Pd core–shell NPs differed after a 1 hour exposure to 375 °C. EDX mapping after heating under H_2_ (10% balanced in Ar) shows uniform distribution of Au and Pd within each particle (Fig. 2a). The EDX line scans show overlapping Pd and Au signals, which confirms that the alloying process was nearly completed. In contrast, the core–shell structures persisted after 1 hour exposure to 375 °C under O_2_ (10% balanced in N_2_). Both the EDX maps (Fig. 2a) and corresponding line scans illustrate the remaining Pd surface enrichment with the Pd line profile clearly extending outside the Au profile. This suggests that the O_2_ atmosphere partially stabilized the core–shell structure and retarded the alloying process.

In fact, the NPs heated under O_2_ were less alloyed than those heated under H_2_ over a range of heating times (5 to 90 min) and eventually completely alloyed after 12 h of heating. Fig. 2b shows the evolution of the degree of core–shell structure upon heating at different times. A value of 1 represents a core–shell structure and a value of 0 a full alloy. A value smaller than 0 would indicate Au enrichment in the NP surface. The degree of core–shell structure was derived by taking line scans from multiple particles in the EDX maps after heating for different amount of time and dividing the width of the Pd profile and the width of the Au profile. In the core–shell particle, the width of the Pd signal is larger than that of Au. Upon alloying the Pd and Au profile start to overlap. This approach is based on a previously validated methodology^35^ and is further described in the experimental methods. Examples of EDX maps and line scans after different heating times can be found in Fig. S3.† Upon heating, the NPs evolve from a core–shell structure to an alloy, with the degree of core–shell structure decreasing from 1 to 0. The degree of core–shell structure was consistently higher under O_2_ than under H_2_, indicating that the alloying kinetics were slower under O_2_. Due to the limited number of data points and the variation in alloying speed between the individual nanoparticles, the error bars are relatively large, preventing further quantification of the alloying kinetics based on these *ex situ* data. Hence, our *ex situ* results indicate that (1) the gas atmosphere influences the dynamics of the alloying process and (2) the AuPd alloy, which is the thermodynamically stable composition in the whole temperature range,^45^ is eventually obtained in the NP bulk regardless of gas atmosphere.

### Following the surface alloying with time-lapse CO-DRIFTS

**Fig. 3:**
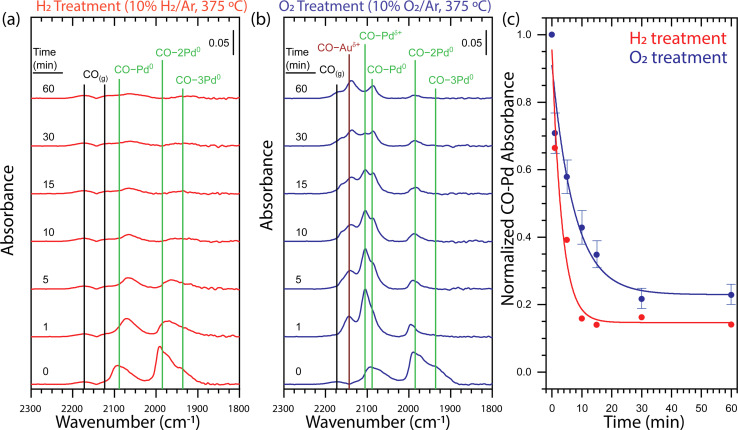
Time-dependent DRIFT spectra for (a) H_2_ and (b) O_2_ treatments of core–shell Au-Pd/SiO_2_ probed by CO adsorption demonstrate that the dissolution of Pd from the surface strongly depends on the gas environment. (c) Integrated CO–Pd bands of *ex situ* time-dependent DRIFT spectra for H_2_ (red) and O_2_ (blue) treatments of core–shell Au-Pd/SiO_2_ normalized at *t* = 0 demonstrate that the H_2_ treatment causes faster Pd dissolution and yields a lower Pd surface concentration. The guidelines through the H_2_ and O_2_ treatment integrated data are fitted with an exponential decay function. The error bars associated to the O_2_ data points originate from the errors in the deconvolution of the CO–Pd peaks, as explained in Fig. S6.† The vibrational feature assignments are tabulated in Table S1† and additional evidence for the assignments are provided in Fig. S5.† Each spectrum panel denotes a 0.05 absorbance intensity legend. H_2_ pretreatment: 5% H_2_ in Ar, 250 °C, 45 min; H_2_ treatment: 10% H_2_ in Ar, 375 °C; O_2_ treatment: 10% O_2_ in Ar, 375 °C; CO adsorption conditions: 0.1% CO in Ar, 25 °C.

To understand the impact of the gas atmosphere on the surface alloying of the core–shell Au-Pd/SiO_2_ nanoparticles, we employed diffuse reflectance infrared Fourier transform spectroscopy measurements using CO adsorption (CO-DRIFTS). First, the samples were pretreated with H_2_ (5% balanced in Ar, 250 °C, 45 min). Then, a series of H_2_ or O_2_ treatments (10% balanced in Ar, 375 °C) followed by a CO treatment (0.1% balanced in Ar, 25 °C) were performed to determine the relative population of adsorbed CO at distinct surface sites as a function of gas atmosphere and time. The surface structure was not affected by prolonged CO exposure (Fig. S4†). In total, the NPs stayed for 1 h at 375 °C and the heating was interrupted to perform CO-DRIFTS. After the H_2_ pretreatment at 250 °C, the surface Pd was fully reduced (Fig. 3a, *t* = 0 min). The DRIFT spectrum was characterized by comparing it to the spectra of an oxidized and reduced Pd/γ-Al_2_O_3_ reference sample using the features at 2173, 2120, 2088, 1985, and 1935 cm^−1^ (Fig. S5†). The features at 2173 and 2120 cm^−1^ were determined to be gas-phase CO (CO_(g)_) by repeating the measurement over a reactor packed solely with SiC. The features at 2088, 1985 and 1935 1935 cm^−1^ were attributed to metallic Pd with CO adsorbed in a linear (CO–Pd^0^), bridging (CO–2Pd^0^), and three-fold hollow (CO–3Pd^0^) configuration, respectively. All assignments of CO adsorbed to metallic Pd are in agreement with the literature.^24,25,46–52^

Sequential thermal treatments in H_2_ at 375 °C over the course of 60 min show rapid Pd dissolution (Fig. 3a). A one-minute H_2_ treatment induced a shift of CO–Pd^0^, CO–2Pd^0^, and CO–3Pd^0^ to lower frequency and a decrease in intensity of all features. A shift to lower frequency of CO bound to metallic Pd is consistent with previous observations attributed to alloying of the metallic Pd surface with Au.^24,47^ An incremental decrease in intensity of CO–Pd^0^, CO–2Pd^0^, and CO–3Pd^0^ was observed up to a 10 minutes H_2_ treatment after which the relatively low intensity of the peaks is unchanging up to 60 minutes. Our observation of an exponential decay in total CO–Pd absorbance is consistent with first-order kinetics for surface Pd dissolution into bulk Au which has been observed on single crystal Pd/Au(111).^24^ The limited ability of CO to adsorb to metallic Au under our CO adsorption conditions^49^ cannot provide direct evidence of an Au-rich surface. However, the decrease in total CO–Pd absorbance from 100 to 15% is consistent with a transition from a Pd- to Au-rich surface and occurs within the first 10 minutes of thermal treatment (Fig. 3c).

A one-minute O_2_ treatment (10% balanced in Ar, 375 °C) of the Au–Pd core–shell sample induced the appearance of two features at 2105 and 2143 cm^−1^, indicating Pd oxidation (Fig. 3b). The feature at 2105 cm^−1^ was attributed to cationic Pd with CO adsorbed in a linear (CO–Pd^*δ*+^) configuration by comparison to an oxidized Pd/γ-Al_2_O_3_ sample^51–53^ (Fig. S5b†). The feature at 2143 cm^−1^ could be attributed to cationic Pd or cationic Au (CO–Au^*δ*+^) with CO adsorbed in a linear configuration but the disappearance of the feature during purging of CO by Ar confirm the assignment as cationic Au (Fig. S5c†).^54^ Sequential O_2_ treatments of the initial Au-Pd core–shell nanoparticle demonstrate Pd dissolution during the transformation of the surface from metallic Pd to a mixed metallic and oxidized AuPd alloy (Fig. 3b). A one-minute O_2_ treatment induced a decrease in intensity of the CO–Pd^0^, CO–2Pd^0^, and CO–3Pd^0^ bands and appearance of the CO–Pd^*δ*+^ and CO–Au^*δ*+^ which indicated oxidation of the material. There was no notable shift in the frequency of the CO bound to metallic Pd which could suggest limited alloying in metallic Pd domains. Incremental O_2_ treatments up to 60 minutes led to a decrease in intensity of all bands. The most notable decrease with respect to the other bands was the relative amount of CO–Pd^*δ*+^.

We find clear differences in the CO-DRIFT spectra obtained upon thermal treatment in H_2_ and O_2_, providing insight in the redistribution kinetics and final surface composition of the AuPd NPs. Fig. 3c shows a comparison of the normalized Pd–CO bands under H_2_ or under O_2_, which was obtained by integrating the total area of the CO bands related to Pd. To separate the contribution of the CO–Au^*δ*+^ peak from the CO–Pd peaks, we performed a Gaussian deconvolution, as demonstrated in Fig. S6.† Under H_2_, the CO absorbance on Pd showed a rapid exponential decay that stabilized after 10 minutes of thermal treatment at ∼15% the initial CO absorbance. On the other hand, under O_2_, the CO adsorption on Pd shows a two times slower exponential decay, stabilizing after 30 min of thermal treatment with ∼23% of the initial CO absorbance. Thus, thermal treatment in H_2_ led to faster Pd dissolution and a- Au-rich surface, whereas the Pd dissolution in O_2_ was considerably slower and a higher concentration of Pd remained at the nanoparticle surface.

The effect of the gas-phase (H_2_ or O_2_) on the final surface termination of the Au-Pd NPs was further investigated using DFT calculations. In the calculations, the subsurface layers consisted of 36 atomic% Pd randomly distributed in Au, similar to our experimental system, and only the composition of the surface layer was varied. We considered four different surface layers: full Au (Au_16_), full Pd (Pd_16_), 6.25% Pd substituted in Au (Au_15_Pd_1_), and 12.5% Pd substituted in Au (Au_14_Pd_2_) (Fig. S7a†). On these surfaces either a single H_2_ or O_2_ molecule was adsorbed. For the Pd-containing surfaces, adsorption of H_2_ and O_2_ always occurred on the Pd-site. On the Au_16_ surface adsorption took place on a Au-site. The full set of adsorption configurations are depicted in Fig. S8.† Note that the effect of subsurface Pd on the adsorption energies was negligible in comparison with the adsorption energies on the surface Pd (Fig. S9†).

The adsorption energies 
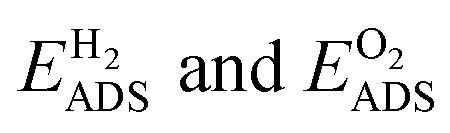
 for the four different surface compositions are shown in (Fig. S7b†) and become more negative with an increasing Pd surface fraction, indicating stronger binding on a Pd-enriched surface. The minimum Pd chemical potential *μ*_Pd_ was found for the Au_15_Pd_1_-surface in O_2_ (Fig. S7c†). O_2_ adsorption on this Pd-monomer surface results in a *μ*_Pd_ that is 0.12 eV lower than on the Au_14_Pd_2_-surface containing a Pd-dimer. Interestingly, the difference between the Au_15_Pd_1_ and Au_14_Pd_2_-surface is much smaller in H_2_, only 0.02 eV. Thus, although Au_15_Pd_1_-surface has a minimal *μ*_Pd_ in both O_2_ and H_2_, the driving force for Pd surface segregation is larger in O_2_. Additionally, the DFT results indicate that Pd monomer formation is energetically favored. Both findings are consistent with the CO-DRIFTs results in Fig. 3, indicating a 15% Pd monomer concentration upon thermal treatment in O_2_. The DFT results are also in line with previous theory studies indicating that Pd oxide formation stabilizes Pd at the nanoparticle surface under O_2_.^27^

### Direct visualization of nanoparticle bulk alloying with *in situ* gas-phase STEM

To directly visualize the alloying process in the Au-Pd NPs, *in situ* STEM experiments under atmospheric pressure of 10% H_2_ or 10% O_2_ in Ar were performed. The NPs were heated to 375 °C for a total time of 60 minutes and were cooled to room temperature at 18 different time steps to image. Cooling to room temperature for imaging allowed us to pause the alloying process and image several regions in the sample. This approach has previously been used for *in situ* heating TEM of Au-Ag NPs in vacuum.^36,37,55^ Here, we followed the alloying process of 25 NPs. Since fast alloying (<10 minutes) was expected based on the CO-DRIFTS results, short heating steps were chosen in the first 15 minutes of the experiment. Fig. S10† depicts the temperature profile and Fig. S11† shows field of view of the regions followed through the experiment under H_2_. The NPs were stable and did not move over the SiN window. Only the few NPs that were very close together sintered during the pretreatment.

**Fig. 4:**
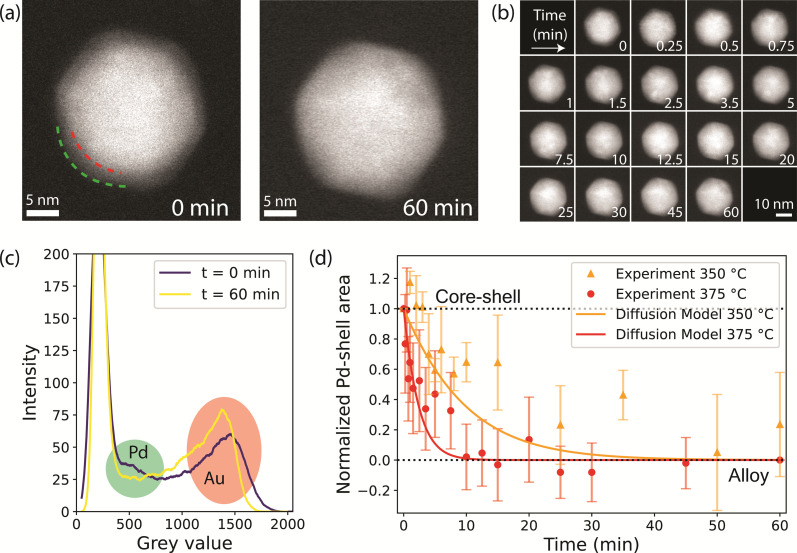
*In situ* STEM visualizing the alloying of Au-Pd core–shell NPs in H_2_ (10% H_2_ in Ar, 375 °C). (a) Example HAADF-STEM image of a NP at *t* = 0 min (left), with the red and green dashed lines highlighting the interface between the Au-core and the Pd-shell and the nanoparticle surface, respectively, and at *t* = 60 min (right), with the alloying process completed. (b) Images of the same NP obtained at different total time (min) at 375 °C. (c) Grey-value histograms of the NP in (a) at *t* = 0 (purple) and *t* = 60 minutes (yellow), with the Pd and Au-related grey-values marked in green and red, respectively. (d) Normalized Pd-shell area decrease in a 10% H_2_ in Ar atmosphere of 1 bar at 350 °C (orange circles) and 375 °C (red circles), indicating a shift from a core–shell structure to an alloy. The error bars represent the standards deviation of the Pd shell related area across measurements of multiple nanoparticles. The solid lines show the alloying kinetics over time calculated from Fick's diffusion law model at 350 °C (orange) and 375 °C (red). The diffusion coefficients were calculated from literature values (see Methods) and at *t* = 0 they are 3.18 × 10^−21^ m^2^ s^−1^ and 1.30 × 10^−20^ m^2^ s^−1^ for the diffusion of Pd into Au, and 1.73 × 10^−26^ m^2^ s^−1^ and 1.11 × 10^−25^ m^2^ s^−1^ for the diffusion of Au into Pd, at 350 and 375 °C, respectively.

Example images of a Au-core Pd-shell NP at *t* = 0 min and of the corresponding alloyed Au-Pd NP at *t* = 60 min are shown in Fig. 4a. The full set of images for this particle is shown in Fig. 4b, revealing the evolution of the NP during the different time steps in an H_2_ atmosphere at 375 °C. The alloying dynamics were monitored by quantifying the disappearance of the Pd-shell in the HAADF-STEM images. Specifically, this was done by determining the loss of the grey value intensities corresponding to the Pd-shell. A core–shell structure, as shown in Fig. 4a, taken at *t* = 0 min, has three regions with distinct grey values: the Au-core in white, the Pd-shell in grey, and the background in black. This contrast predominantly originates from the atomic weight difference between Au and Pd. To illustrate this, the grey-value histograms for each region are shown in Fig. S12.† In the histogram of a NP, the Pd related grey values show as a shoulder between the darker pixels belonging to the background and the brighter pixels belonging to Au (Fig. 4c).

Upon alloying, the Pd-shell disappears, which leads to a decrease in the number of pixels corresponding to Pd in the histogram. An example set of histograms for one NP is shown in Fig. S13,† where the highlighted region indicates the grey values associated with the Pd-shell. It is important to note that apart from Z-contrast between Au and Pd, there is also thickness contrast, that intrinsically leads to darker pixels at the NP edges even when the metal distribution is uniform. To account for this, we use the histogram at *t* = 60 min as a baseline and compute the area between the histogram lines at each time point and the final state (*t* = 60 min) for the grey values that correspond to the Pd-shell. A detailed explanation of the method is found in the ESI† (Fig. S12–S15†). To validate our data analysis approach, we performed an *in situ* heating experiment under vacuum following the alloying process both by EDX (as previously performed in ref. 35) and HAADF-STEM imaging. This allowed us to benchmark the results obtained from the new HAADF-STEM data analysis methodology with an already established method.^35^ The degree of alloying *vs.* temperature data in Fig. S16† confirm that the results were very similar and that our HAADF-STEM analysis method can be used to reliably quantify the alloying dynamics.

Fig. 4d shows the decrease in Pd-shell area averaged over all 25 particles as a function of time during thermal treatment in H_2_ at 375 °C. The data indicate that the alloying was complete within 10 minutes, closely matching our CO-DRIFTS results. We verified that the alloying kinetics derived from the *in situ* TEM data were not impacted by electron beam irradiation. To do so, a total of 20 NPs were imaged less frequently during the same experiment, thereby accounting for less than half of the electron dose. The Pd-shell grey value area evolved in the same manner as for the more irradiated NPs (Fig. S17†). Furthermore, EDX showed fully alloyed NPs in both irradiated and non-irradiated areas (Fig. S18†). These results therefore proof that the electron beam did not substantially influence the alloying dynamics.

**Fig. 5:**
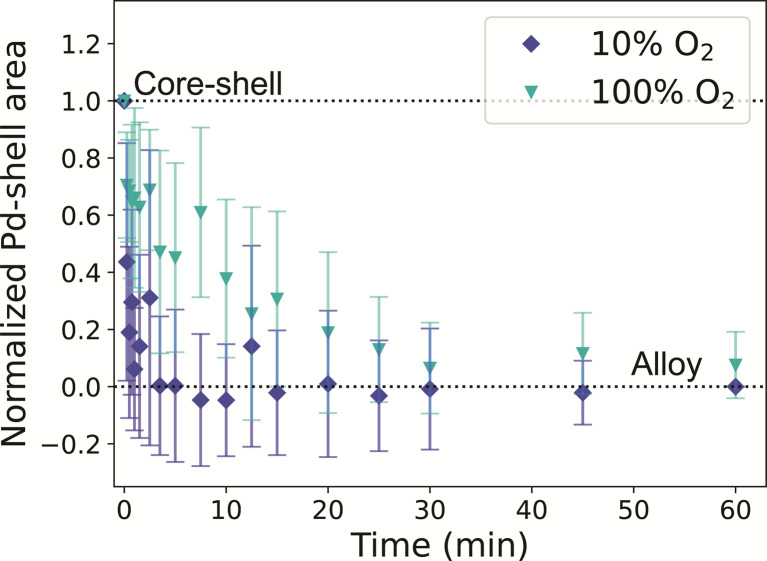
*In situ* STEM visualizing the alloying of Au-Pd core–shell NPs in O_2_ at 375 °C. The plot shows the normalized Pd-shell area as a function of time for a 10% O_2_ in Ar (dark blue) and 100% O_2_ (light blue) gas atmosphere. The error bars represent the standard deviation of the Pd shell related area across measurements in multiple nanoparticles.

When the same experiment was performed under 10% O_2_, the normalized Pd-shell related area in the histograms decreased in slightly faster than in our H_2_ experiments (Fig. 5a and S19†), and the alloying process seemed to be completed after 5 minutes. After 60 minutes at 375 °C, the NPs were fully alloyed, both in irradiated and non-irradiated regions (Fig. S20†). The alloying under O_2_*in situ* occurred faster than expected based on our *ex situ* experiments. We investigated if this discrepancy could be attributed to electron beam irradiation. Consistent with the H_2_ experiment, regions imaged with less than half of the dose evolved in a similar way compared to NPs that received a higher electron dose (Fig. S19b†). To further investigate the possibility of electron beam effects, the experiment was repeated without beam irradiation following the same heating profile as used in the *in situ* TEM measurements. EDX after thermal treatment confirmed that all NPs were fully alloyed (Fig. S21†), and hence no indications for the beam effects altering the alloying process. However, the alloying kinetics did depend on the oxygen pressure used. When repeating our *in situ* TEM measurement with a higher partial pressure of oxygen (100%) the alloying process slowed down, and the nanoparticles alloyed after 30 minutes, coinciding with our CO-DRIFTS results (Fig. 5). Hence, a higher partial pressure of O_2_ was needed in the *in situ* TEM setup to observe the effect of the O_2_ seen in the CO-DRIFTS and *ex situ* TEM experiments.

We also investigated the temperature effect on the alloying dynamics by performing an additional experiment in H_2_ at 350 °C. The Pd-shell related area decreased significantly slower compared to the experiment at 375 °C (Fig. 4d), indicating substantially slower alloying. This result demonstrates that (1) the temperature significantly affects the alloying dynamics of the NP bulk and that (2) our data analysis methodology is capable of detecting significant speed differences in the alloying process. Interestingly, our data can be described well by a numerical model based on Fick's second law. In short, the model is used to calculate the amount of Au and Pd atoms passing through a Au–Pd interface at each time point, and has previously been applied to describe the alloying of Au-Ag nanorods.^35^ The diffusion coefficients were calculated with bulk data of the frequency factor (*D*_0_) and activation energy (*Q*),^56^ corrected for nanoparticle finite-size effects, which account for a ∼4% decrease in *Q*.^35,57^ The model relies on iterating the calculation of the quantity of atoms that diffuse at each time step, followed by updating of the composition of the interface. Using this model, we find that the diffusion coefficients at *t* = 0 are 3.18 × 10^−21^ m^2^ s^−1^ and 1.30 × 10^−20^ m^2^ s^−1^ for the diffusion of Pd into Au, and 1.73 × 10^−26^ m^2^ s^−1^ and 1.11 × 10^−25^ m^2^ s^−1^ for the diffusion of Au into Pd, at 350 and 375 °C, respectively. The modeled diffusion behavior at 350 and 375 °C is shown in Fig. 4d, and it shows a good agreement with datapoints derived from the *in situ* TEM data both at 350 and 375 °C. This validates our *in situ* TEM approach, and it also shows that the modeled diffusion is accurate for nanoparticle systems.

## Discussion

CO-DRIFTS showed that the final surface composition of the Au-Pd nanoparticles is highly affected by the gas atmosphere, and that Pd is, in part, stabilized at the surface after thermal treatment in an O_2_ atmosphere. Under H_2_, after 1 h at 375 °C, the Au-Pd NPs surface was Au-rich and 85% of the Pd had diffused to the bulk of the NPs. Contrarily, under O_2_, the surface still consisted of a mixture of Au and oxidized Pd. While Pd segregation into the bulk has also been observed under O_2_, it was less prominent than under H_2_, with a loss of 77% of the original surface Pd. These findings are consistent with literature reporting on the gas dependent segregation effects in Au-Pd alloy nanoparticles. Similar to our work, Pd-enriched surfaces have been observed after thermal treatment in O_2_ (ref. 30) whereas Au-rich surfaces have been reported for thermal treatment in H_2_ (ref. 25) or vacuum (ref. 23). Experimental EXAFS and XPS results have shown that Pd is oxidized under O_2_, and catalytic testing confirmed that O_2_ treated Au-Pd NPs are more active in hydrogenation and oxidation reactions than H_2_ treated Au-Pd NPs, possibly due to Pd surface enrichment upon O_2_ treatment.^27,58^ Thus, our findings demonstrating the difference in Au–Pd surface composition upon H_2_ and O_2_ treatment are in good agreement with previous studies on dilute Au-Pd nanoparticle systems (<10 atomic% Pd) and confirm that the same trends hold for higher Pd-content Au–Pd systems.

Our time-lapse CO-DRIFTS measurements (Fig. 3) allowed direct assessment of the Pd surface segregation kinetics in O_2_ and H_2_. Under H_2_, Pd quickly segregated to the NP bulk and the surface composition stabilized after 10 minutes at 375 °C. Under O_2_, the Pd segregation kinetics were two times slower, and a stable composition only reached after 30 minutes at 375 °C. Thus, Pd dissolution was directly affected by the gas atmosphere, where Pd stabilization at the surface affects its dissolution kinetics into the nanoparticle bulk. Our *ex situ* TEM experiments (Fig. 2) validate the CO-DRIFTS results and confirm that the alloying happens slower under O_2_ than under H_2_. The differences in time scales for surface Pd dissolution may be attributed to the higher surface sensitivity of CO-DRIFTS, which only analyzes the composition of the outermost atomic layer. To the best of our knowledge, only one study has previously provided dynamic measurements on a Pd/Au (111) single crystal model systems,^24^ but this had never been extended to Au–Pd nanoparticle systems. Our observation of an exponential decay in total CO–Pd absorbance is consistent with first-order kinetics for surface Pd dissolution into bulk Au observed on single crystals,^24^ suggesting a similar Pd-dissolution kinetic behavior between single crystals and nanoparticles. Our DFT calculations correctly predict incomplete Pd dissolution from the surface in both gas atmospheres, with more Pd stabilization under O_2_, in line with our CO-DRIFTS results and previous DFT studies.^27^ Moreover, the chemical potential is the lowest for Pd monomers, indicating their stabilization at the surface. This stabilization of the Pd monomers is indeed observed in the CO-DRIFTS, where the CO–Pd peak disappears slower when compared to the CO–2Pd or CO–3Pd peak. The stabilization of surface Pd after O_2_ treatment likely leads to enhanced catalytic activity due to Pd surface enrichment.^27^

Moreover, we directly visualized and quantified the alloying dynamics in the bulk of core–shell bimetallic NPs in gas-phase through *in situ* TEM for the first time by following the changes in the grey value histograms. Previous studies have mainly used aberration corrected environmental TEM to visualize the loss^20^ or the formation^21^ of a core–shell structure. Studies on the dynamics of the alloying process upon heating have been performed under vacuum, using EDX,^35^ fast electron tomography,^36,37^ or atomic resolution imaging.^38,39^ These approaches offer a more precise quantification method but are difficult to perform under atmospheric pressure of gas. The main limitation of our data analysis method is the large deviation in the Pd-shell related area among nanoparticles (see error bars in Fig. 4 and 5), which restricts drawing conclusions for small differences in alloying dynamics among nanoparticles. The element-specific Z-contrast in HAADF-STEM mode is complicated by the additional thickness- and diffraction-based contrast, which makes it more difficult to quantify the loss in Pd-shell contrast from our 2D STEM images. The thickness dependent contrast causes the edges of the NP to look darker than the center. On the other hand, the diffraction contrast broadens and displaces the white peak in the histogram. While we correct for the thickness contrast, the Pd-shell related area does not always follow the expected decreasing behavior at individual NPs, resulting in relatively large error bars. We validated our methodology in vacuum and show that the results obtained from the HAADF-STEM data analysis closely match the results obtained from EDX when imaging under vacuum (Fig. S16†). Furthermore, our methodology does not only allow acquiring images at atmospheric pressure, but also drastically reducing the electron dose and acquisition time, especially when compared to EDX mapping. Reducing the imaging time allows imaging at more time points or at different regions of the sample, allowing the *in situ* monitoring of many different particles within the same experiment.

The effect of the gas atmosphere (H_2_ or O_2_) on the Au–Pd alloying dynamics observed in our *in situ* TEM experiments was smaller than the effect of different temperatures (350 *vs.* 375 °C). Under 10% H_2_, the alloying process was completed after 10 minutes, matching our CO-DRIFTS results (Fig. 4). However, the expected slowdown of the alloying process did not occur under 10% O_2_, but only happened when using higher oxygen pressures (100%). The alloying process was significantly slowed down under 100% O_2_, and the process was completed after 30 minutes, coinciding with our CO-DRIFTS results (Fig. 5). This likely indicates that a higher partial pressure is needed during the *in situ* TEM measurements to allow the formation of the Pd oxide to stabilize Pd on the surface of the NPs, and hence retard the alloying. Indeed, the formation of a Pd oxide has been observed *in situ* in Au-Pd nanoparticles under 1 atm O_2_.^59^

The alloying kinetics derived from our *in situ* TEM results closely match our model based on Fick's diffusion model and show that a decrease in temperature of 25 °C leads to a pronounced slowdown in alloying dynamics. This diffusion model has previously been validated for Au-Ag core–shell nanorods of ∼70 nm length.^35^ Furthermore, for Au-Ag NPs at 450 °C, the diffusion simulation yields diffusion coefficients with the same order of magnitude to those previously observed experimentally.^36,37^ With this *in situ* study, we show that the alloying behavior of Au-Pd NPs is also described well with this model, when considering the physical properties of the metals and a correction for NP size and shape. We therefore expect that this diffusion model is able to predict alloying dynamics in a whole range of metals, temperatures, and nanoparticle sizes. Given the superior catalytic activity of Au-Pd core–shell nanoparticles with respect to their alloyed counterparts,^17^ this model can help in designing the catalytic testing conditions such that alloying and the associated loss in catalytic activity do not occur during the reaction.

## Conclusion

Our combined CO-DRIFTS and *in situ* TEM study highlights the crucial role of the gas atmosphere on the surface alloying of Au-Pd NPs, and its more limited impact on the alloying dynamics of the NP bulk. Exposure to H_2_ at 375 °C led to faster Pd dissolution from the surface than under O_2_ showing that Pd was partially stabilized at the NP surface in an O_2_-rich atmosphere. The DFT-calculations indicate that this can be ascribed to a lower chemical potential for Pd-monomers at the surface under an O_2_ atmosphere. *In situ* TEM allowed the direct visualization of the alloying dynamics in the nanoparticle bulk and showed that the diffusion in the NP bulk is mostly impacted by the heating temperature, and to a certain extend also by the partial pressure of O_2_. Through our data analysis methodology, we were able to extract the alloying kinetics from the HAADF-STEM images allowing for short imaging times, low electron doses, and for a large number of nanoparticles followed at multiple time steps. The experimentally attained diffusion kinetics were captured well by a numerical model based on Fick's second law of diffusion, and we expect that this model can be translated to other nanoparticle systems as well. Altogether, our work introduces a new approach to quantitatively assess the alloying dynamics in bimetallic nanoparticle systems through *in situ* TEM in reactive gasses and presents new insights into gas dependent segregation phenomena on surface and bulk alloying of multi-metallic nanoparticle systems.

## Experimental section

### Chemicals

Trisodium citrate dihydrate (SC, ≥99.0%), tannic acid (TA), potassium carbonate (K_2_CO_3_, ≥99.0%), chloroauric acid (HAuCl_4_, 99.9%), sodium tetrachloropalladate (ii) (Na_2_PdCl_4_, 98%) and polyvinylpyrrolidone (PVP, MW 55 000) were obtained from Merck. Aerosil OX 50 was purchased from Degussa. Correl-like activated carbon was purchased from Norit. Ultrapure water with a resistivity of 18.2 mΩ cm^−1^ (Millipore Milli-Q grade) was used. All chemicals were used without further purification. The glassware used for AuNP synthesis and Pd overgrowth was cleaned overnight with aqua regia (HCl/HNO_3_ mixture in a 3 : 1 ratio by volume), rinsed thoroughly with Milli-Q water and dried in an oven.

### Au NP synthesis

Gold nanoparticles of around 20 nm in diameter were synthesized following the procedure of Piella *et al.*^42^ Briefly, a solution of 450 mL of sodium citrate 2.20 mM, 0.30 mL 2.50 mM tannic acid and 3.00 mL 150 mM potassium carbonate were heated to 70 °C while stirring vigorously. Then, 3.00 mL 25.0 mM HAuCl_4_ were added, and the temperature was maintained for 10 minutes. Within 3–5 min, the color changed from pale yellow to red, indicating the formation of small gold nanoparticles, and the pH shifted from 10 to 8. Then, 165 mL of this seed solution were extracted, and 165 mL of 2.20 mM sodium citrate solution were added. When the temperature reached 70 °C again, 1.50 mL 25.0 mM HAuCl_4_ were injected twice with a 10 minutes interval. This process was repeated for an additional 8 times, until a total of 30.0 mL 25.0 mM HAuCl_4_ had been added. After the last step, the reaction mixture was allowed to cool down and stored in the dark, and the UV-vis spectra of each growth cycle was measured.

### Pd overgrowth

For the Pd overgrowth, 1.50 mL polyvinylpyrrolidone solution (1 g/10 mL H_2_O, Mw = 55 000 g mol^−1^) were added to 150 mL of the as-synthesized sodium-citrate-capped gold nanoparticles and stirred overnight. The pH was adjusted to 4 with 0.10 M HCl (2.50 mL) to ensure a slow enough reaction rate during the Pd overgrowth.^5^ Next, 7.80 mL Na_2_PdCl_4_ 10 mM were added to the solution and it was stirred for 5 minutes before rapidly adding 7.80 mL of 40 mM ascorbic acid under vigorous stirring to ensure homogeneous reduction of the Pd precursor onto the Au-cores. The solution was left stirring overnight at 400 rpm. Afterwards it was centrifuged at 12 000 rcf for 1 h and kept in half the volume of water for storage in the fridge.

### Deposition on a silica support

200 mg of (Aerosil OX50) were mixed with 10 mL EtOH and sonicated for 15 minutes to ensure proper dispersion. Half of the synthesis volume of the Au-Pd NPs was centrifuged and resuspended in 20 mL EtOH and added to the silica suspension. After sonicating for approximately one hour, the purple dispersion was divided in two 50 mL centrifuge tubes, and 15 mL of toluene was added to each tube as an antisolvent, leading to homogeneous deposition of the AuPd NPs on the silica support.^17^ Then, it was centrifuged at 2000 rcf for 5 minutes and the clear supernatant was removed. The pellet was dried in an oil bath at 60 °C overnight. To remove the ligands, the supported AuPd/SiO_2_ NPs were heated in a U-shaped reactor with a ramp of 2 °C per minute and a flow of 100 mL min^−1^ of 10% O_2_ balanced in N_2_ and were kept at 300 °C for 3 hours. This resulted in a 4.7 wt% of metal on the Aerosil OX50 support.

### Thermogravimetric analysis (TGA)

Thermogravimetric analysis (TGA) was performed on a TA Instruments Discovery TGA5500 equipped with an IR furnace and hyphenated with a MKS Cirrus 3 mass spectrometer (MS). About 2–4 mg of sample were loaded in the reactor. The samples were first heated to 110 °C to remove adsorbed water. Then, they were heated up to 600 °C in a flow of 20% O_2_ in Ar, with a ramp of 5 °C min^−1^. The CO_2_ release was monitored by the MS.

#### Inductively coupled plasma (ICP)

Inductively coupled plasma (ICP) was conducted by Mikroanalytisches Laboratorium Kolbe (Oberhausen) on supported AuPd/SiO_2_.

#### Diffuse reflectance infrared Fourier transform spectroscopy (CO-DRIFTS) experiments

DRIFTS experiments were carried out in a low-dead volume reaction chamber (Harrick Scientific) equipped with ZnSe windows, mounted inside the sample compartment of a Bruker Invenio FT-IR spectrometer using a Praying Mantis diffuse reflectance accessory (Harrick Scientific). The gas flow through the DRIFTS reactor was controlled by a rapid switching gas handling system.^60^ The catalyst sample was prepared by pressing and sieving between 304 stainless-steel meshes (50 × 50 mesh and 70 × 70 mesh). The DRIFTS reactor was loaded with approximately 135 mg of 46 grit SiC, an inert support with high thermal conductivity, followed by approximately 15 mg of the prepared AuPd/SiO_2_ catalyst. Large temperature gradients can exist between the thermocouple contact in a DRIFTS reactor cell and the catalyst surface temperature exposed to the infrared beam.^61^ Therefore, the temperature was calibrated by accounting for the thermal gradient between the thermocouple and the catalyst surface by using an optical pyrometer with an emissivity of 0.95. All DRIFTS experiments used a total volumetric flow rate of 50 mL min^−1^. Each absorbance spectrum was obtained by averaging 200 background and sample scans at a resolution of 4 cm^−1^ using a liquid-nitrogen-cooled HgCdTe (MCT) detector, while the Praying Mantis diffuse reflectance accessory and FT-IR spectrometer was purged with dry N_2_. The AuPd/SiO_2_ pretreatment involved reduction under H_2_ (5% H_2_ in Ar, 250 °C) for 45 min. The sequential H_2_/O_2_ treatments (10% H_2_/O_2_ in Ar, 375 °C) involved heating, holding for the denoted time and cooling in the H_2_/O_2_ environment. CO-DRIFTS measurements were taken after 0, 1, 5, 10, 15, 30 and 60 minutes (total time at 375 °C). The sample measurements were acquired at 25 °C once the saturation of adsorbed CO in a 0.1% CO in Ar was achieved. Quantitative analysis of DRIFTS spectra can be prone to errors because adsorbate concentration is truly neither proportional to absorbance intensity nor Kubelka–Munk (KM)^62,63^ intensity but can be nearly proportional to one intensity unit depending on the relative reflectivity of the DRIFTS features.^64^ The high relative reflectivity of our DRIFTS features demonstrate the integration of absorbance intensity is expected to be nearly proportional to concentration while Kubelka–Munk intensity is less appropriate (Fig. S22†).^64^

### *Ex situ* heating experiments

Around 5 mL of AuPd NPs were centrifuged and resuspended in EtOH and then drop-casted onto a carbon coated TEM grid (H_2_ experiments) or a SiN TEM chip (O_2_ experiments). Prior to heating, the PVP ligands were removed by washing the grid with activated carbon.^43^ In a small beaker, activated carbon was mixed with ethanol. When the bubbling stopped, the grid was submerged for 10 minutes and then let dry. The grid was heated in a tubular oven (Thermolyne 79 300 tube furnace) at 375 °C under 10% H_2_ in Ar or in a U-shaped reactor under 10% O_2_ in N_2_. The heating rate was 5 °C min^−1^ and the gas flow was constant at 100 mL min^−1^. The dwell time at 375 °C varied from 5 min to 12 h in the different experiments.

The degree of core–shell structure in Fig. 2c was calculated by a procedure similar to literature.^35^ At each time point, a minimum of 8 line scans (30 × 5.5 nm) from different nanoparticles were taken from the EDX maps, to get some statistics on the distribution of Au and Pd within the nanoparticle. Note that after oven heating, particles that were close to each other aggregated. The line scans used for analysis were obtained from non-aggregated particles, or taken in the direction where the particles did not touch each other. The degree of core–shell structure was calculated by the division of the full width at half maximum of Au by that of Pd in the line scans. It was then normalized so that the degree of core–shell structure is 1 for the core–shell structure, and 0 when the alloying is complete. Note that it is the same procedure than the one in ref. 35 for the degree of alloying, but the normalization process is different.

### Transmission electron microscopy

Transmission electron microscopy (TEM) was performed on a Talos F200X (Thermo Fisher Scientific) operated at 200 kV. Particle size distributions were obtained by measuring 100 to 200 nanoparticles from bright field TEM images. First, a threshold was applied to mask the nanoparticles, and the size was analyzed using the “analyze particles function” in ImageJ.^65^ For energy-dispersive X-ray (EDX) mapping, the microscope was operated in scanning-TEM (STEM) mode, with a screen current of 1.18 nA and a dwell time of 2–5 μs per pixel, a pixel size of 0.3827 nm, 512 × 512 pixels per image and a camera length of 98 mm. The total time of an EDX map was of 15 minutes. The Super-XTM EDX detector present in the microscope was used to collect the EDX signal and it was quantified using the Velox software. The EDX maps were prefiltered by averaging 5 pixels before quantification. Both Au and Pd were quantified using the L-lines. High resolution electron microscopy was acquired with a double aberration corrected Spectra 300 (Thermo Fisher Scientific) with 2048 × 2048 pixels of 13.6 pm pixel size, a dwell time per pixel of 2.5 μs, a screen current of 0.42 nA, camera length of 90 mm and convergence angle of 30 mrad.

### *In situ* gas-phase scanning transmission electron microscopy

The *in situ* gas-phase STEM measurements were performed using a Protochips Atmosphere system and STEM/EDX optimized chips on a Talos F200x. The chips were glow discharged for 90 s with 10 V of tension. Then, several droplets of Au-Pd NPs dispersed in ethanol were drop-casted on the top chip. The chip was washed with the activated carbon method described in the previous section and let dry before assembling the holder. Once in the microscope, the holder was pump-purged three times to 0.5 mbar to remove air from the system and leak checking was performed. Then, the sample was heated to 250 °C in 5% H_2_ in Ar for 45 minutes to ensure the removal of water from the system and reduction of potentially oxidized Pd species, and cooled down to room temperature. For the experiments under oxygen atmosphere, the holder was pump/purged with Ar 10 times to 20–200 mbar to ensure H_2_ removal and to avoid its mixture with O_2_. During the experiment, the gasses (10% H_2_ or O_2_ diluted in Ar or 100% O_2_) were introduced at 1 bar with a 0.1 sccm flow rate. Then, the sample was heated to 375 °C for a total time of 1 h. The heating was interrupted after 0.25, 0.5, 0.75, 1, 1.5, 2.5, 3.5, 5, 7.5, 10, 12.5, 15, 20, 25, 30, 45 and 60 minutes, and the imaging was performed at room temperature at 4 or 5 different regions, accounting for 29 NPs under 10% H_2_ and 31 NPs under 10% O_2_ and 33 NPs under 100% O_2_. To study the effect of the electron beam dose, some regions were only imaged after 1, 5, 15, 30, 45 and 60 minutes in the 10% H_2_ and O_2_ experiments. The temperature ramp was always 10 °C s^−1^ and the beam was always blanked at high temperature. The imaging was performed at room temperature in HAADF-STEM mode, camera length of 125 mm, 2048 × 2048 pixels with 0.09567 nm pixel size, a dwell time of 5 μs per pixel, a screen current of 25 pA, accounting for a dose rate of 41.3 e^−^ (A^2^ s)^−1^. Moreover, 20 images taken with 100 ns dwell time per pixel were taken and put together using a DCFI algorithm in VELOX. The total dose was 15 570 e^−^ A^−2^ for the regions with 18 heating steps and 6055 e^−^ A^−2^ for the regions with 7 heating steps. After the experiment, the chips were disassembled and kept for inspection and EDX mapping under vacuum. An extra experiment was performed at 350 °C, where after the pretreatment, the heating was interrupted after 1, 2, 3, 4, 5, 6, 8, 10, 25, 35, 50 and 60 minutes. A total of 13 NPs were followed through the experiment. To ensure complete alloying after the experiment, the NPs were heated to 450 °C for 5 minutes and imaged afterwards. The temperature plots of the experiments are shown in Fig. S10.†

### *In situ* heating transmission electron microscopy (under vacuum)

The *in situ* heating TEM experiments under vacuum were performed using a DENS solutions Wildfire heating holder, in a similar fashion as in literature.^35^ The sample was drop-casted on the chip, and was heated step-wise from room temperature to 600 °C with steps of 50 °C. At each temperature, an EDX map and several HAADF-STEM images were taken.

### Data analysis

Extended information of the methodology of the *in situ* STEM data analysis is presented in the ESI.†

### Diffusion model

A numerical diffusion model based on the approximation of Fick's second law of diffusion was used to describe the alloying behavior of the Au-core Pd-shell nanoparticles. This model has previously been used and validated for the alloying in Au-core Ag-shell nanoparticles.^35^ Briefly, the numerical calculations to compute the number of Pd and Au atoms diffusing through the Au–Pd interface per time step at a specific temperature using the following formula (eqn (1)):1
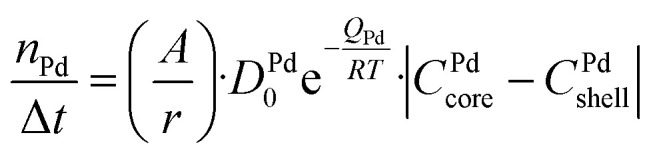
where *A* is the interface area, *r* the radius of the NP, *D*_Pd_ the frequency factor, *Q*_Pd_ the activation energy, *R* the gas constant, *T* the temperature, *C*^Pd^_core_ core the Pd concentration of the core and *C*^Pd^_shell_ shell the Pd concentration of the shell. The bulk frequency factor *D*^Pd^_0_ and activation energy *Q*_Pd_ were taken from literature and were 0.2 cm^2^ s^−1^ and 47 kcal mol^−1^, respectively.^56^ An analogous formula was used for the diffusion of Au through the interface, with frequency factor *D*^Au^_0_ and activation energy *Q*_Au_ 0.12 cm^2^ s^−1^ and 62 kcal mol^−1^, respectively.^56^ The bulk *Q* values were corrected for the NP size effects in the same manner as in literature (eqn (2)), and consist on the calculation of a shape factor (*α*_shape_), described in eqn (3), where *D* is the nanoparticle diameter, *γ*_s_ and *γ*_l_ are the surface energies in the solid and liquid phases (obtained from^22^), respectively, *S* the surface area of the NP, *V* the volume and Δ*H*_m,∞_ the bulk melt enthalpy.^35,57^ After this finite-size effects correction, *Q*_Au_ and *Q*_Pd_ were 59.6 and 45.2 kcal mol^−1^, respectively.2
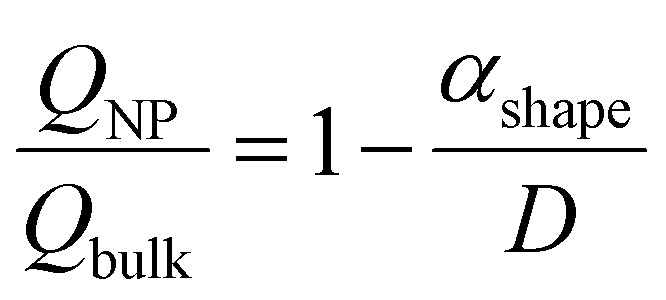
3
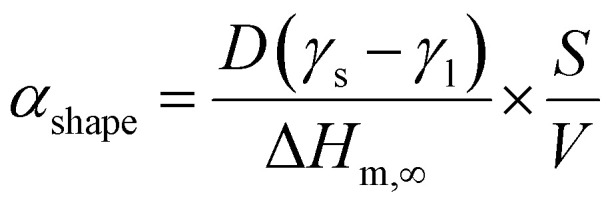


The initial state is a pure Au-core and pure Pd-shell phase connected through an interface. For each time step of 1 s the number of Au and Pd atoms passing through the interface was calculated, and the composition of both sides of the interface was updated. The Pd atoms migrate from the shell to the core and the Au atoms from the core to the shell. These steps were iterated until the fully alloyed state was reached, in which the concentration of Pd in the core was equal to that in the shell. In our case, the calculations were run for *T* = 350 and 375 °C.

### Density functional theory (DFT) calculations

All DFT calculations were performed using the FHI-aims software^66^ and the Perdew–Burke–Ernzerhof (PBE)^67^ generalized gradient approximation (GGA) exchange–correlation functional. Long-range van der Waals interactions were corrected with the Tkatchenko-Scheffler method.^68^ The Pd_0.36_Au_0.64_ composition (suggested by experimental results) was modelled as an 80-atom random alloy in the face-centered cubic (FCC) structure and a lattice constant of 4.076 Å. The (1 1 1) surface was modeled using a periodic slab model with 5 atomic layers and a 4 × 4 (11.54 × 11.54 Å) surface cell. A 16-atom monolayer of pure Au (Au_16_), pure Pd (Pd_16_), monomeric Pd in Au (Au_15_Pd_1_), or dimeric Pd in Au (Au_14_Pd_2_) was added on top of the random alloy surface slab (see Fig. S7a†). A vacuum region with a width of ∼15 Å was introduced to avoid erroneous interactions. For each of the surface models, the bottom 5 atomic layers were frozen at the ideal bulk positions and the top layer was optimized until the forces were converged to a threshold of 0.01 eV Å^−1^. Brillouin zone sampling was done with a Γ-centered 7 × 7 × 1 *k*-point mesh. Geometry optimizations used the Broyden–Fletcher–Goldfarb–Shanno (BFGS) optimization algorithm.^69–73^ Geometry optimizations with FHI-aims employed the predefined ‘light’ settings (4th order expansion of the Hartree potential, radial integration grids with 302 points in the outer shell, and a tier 1 basis set). The structural energies were subsequently refined with the ‘tight’ settings (6th order expansion, 434 grid points, and a tier 2 basis set). Self-consistency calculations were considered converged when the volume-integrated root-mean square change of the charge density between the present and previous self-consistent field iteration was less than 10^−6^ eV Å^−3^ and the total energy changed less than 10^−6^ eV.

The adsorption energy *E*_ADS_ (eV) of O and H was calculated as (eqn (4))4
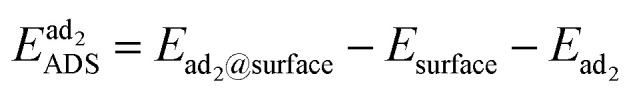
where ad_2_ is either H_2_ or O_2_, *E*_ad_2_@surface_ is the DFT energy of the surface with the adsorbed molecule, *E*_surface_ is the DFT energy of the clean surface, and *E*_ad_2__ is the DFT energy of the gas phase H_2_ or O_2_ molecule.

The chemical potential *μ*_Pd_ (eV) of the Pd surface atoms was calculated as (eqn (5))

5 *μ*^surf^_Pd_ = (*E*^ad^_ADS_{Au_*N*−*n*_Pd_*n*_} − *E*^ad^_ADS_{Au_*N*_})/*n*

where *E*^ad^_ADS_ is the adsorption energy of adsorbate *A* in eV, *N* is the total number of surface atoms and *n* is the number of Pd atoms in the surface. Zero-point energy contributions to the chemical potential were neglected.

## Data availability

The data from the Density Functional Theory (DFT) calculations can be obtained from the GitHub repository at https://github.com/atomisticnet/2024-AuPd-DFT-data.^74^ The dataset contains atomic structures and energies in the formats used by the FHI-aims software.^66^ The raw *in situ* TEM datasets and grey-value histograms can be obtained from the Dataverse repository.^75^ The python code for the analysis of the *in situ* TEM data and Fick's diffusion model is available upon request to the corresponding author.

## Conflicts of interest

There are no conflicts to declare.

## Supplementary Material

TA-012-D4TA03030C-s001
