# Supplementary material for: In situ analysis of gas dependent redistribution kinetics in bimetallic Au-Pd nanoparticles
Source: J Mater Chem A Mater. 2024 Jun 27;12(47):32760–74. doi: 10.1039/d4ta03030c (PMC11627011; doi:10.1039/d4ta03030c)
Supplement: TA-012-D4TA03030C-s001 [file TA-012-D4TA03030C-s001.pdf]

Electronic Supplementary Material (ESI) for Journal of Materials Chemistry A.
This journal is © The Royal Society of Chemistry 2024

Supplementary information: *In Situ* Analysis of Gas Dependent Redistribution Kinetics in Bimetallic Au-Pd Nanoparticles

Marta Perxés Perich[1], Christopher R. O'Connor[2], Koen M. Draijer[1], Nienke L. Visser[1], Nongnuch Artrith[1], Christian Reece[2], Petra E. de Jongh[1] and Jessi E.S. van der Hoeven[1*]

[1] *Materials Chemistry and Catalysis, Debye Institute for Nanomaterials Science, Utrecht University, 3584 CG Utrecht, The Netherlands*

[2] *Rowland Institute at Harvard, Harvard University, Cambridge, Massachusetts 02142, USA*

*E-mail: j.e.s.vanderhoeven@uu.nl

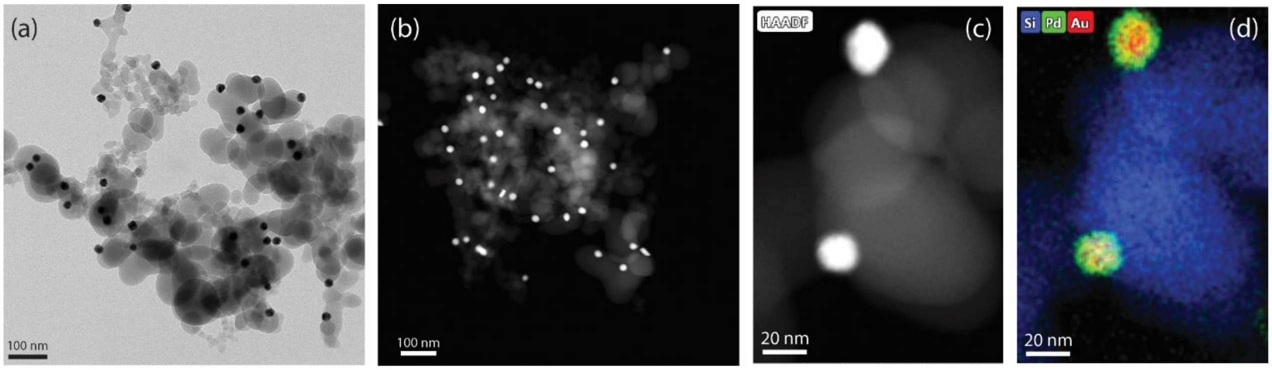

Figure S1. Characterization of silica supported Au-Pd core-shell NPs after calcination at 300 °C. (a) TEM image. (b) HAADF-STEM image. (c) Zoomed-in HAADF-STEM image and (d) its corresponding EDX map demonstrating the core-shell structure.

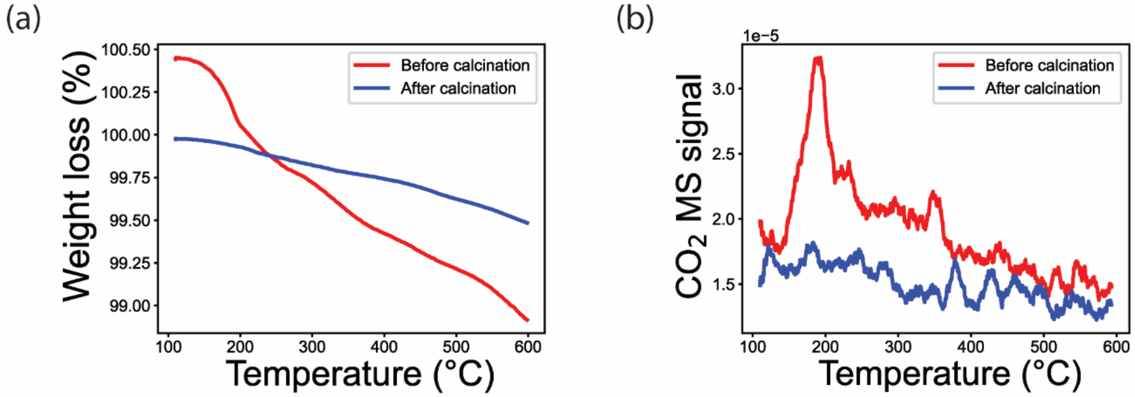

Figure S2. Thermogravimetrical analysis (TGA) of the ligand removal. (a) Weight loss before (red) and after (blue) the calcination at 300 °C (ligand removal step). (b) CO_2 signal in an attached mass spectrometer.

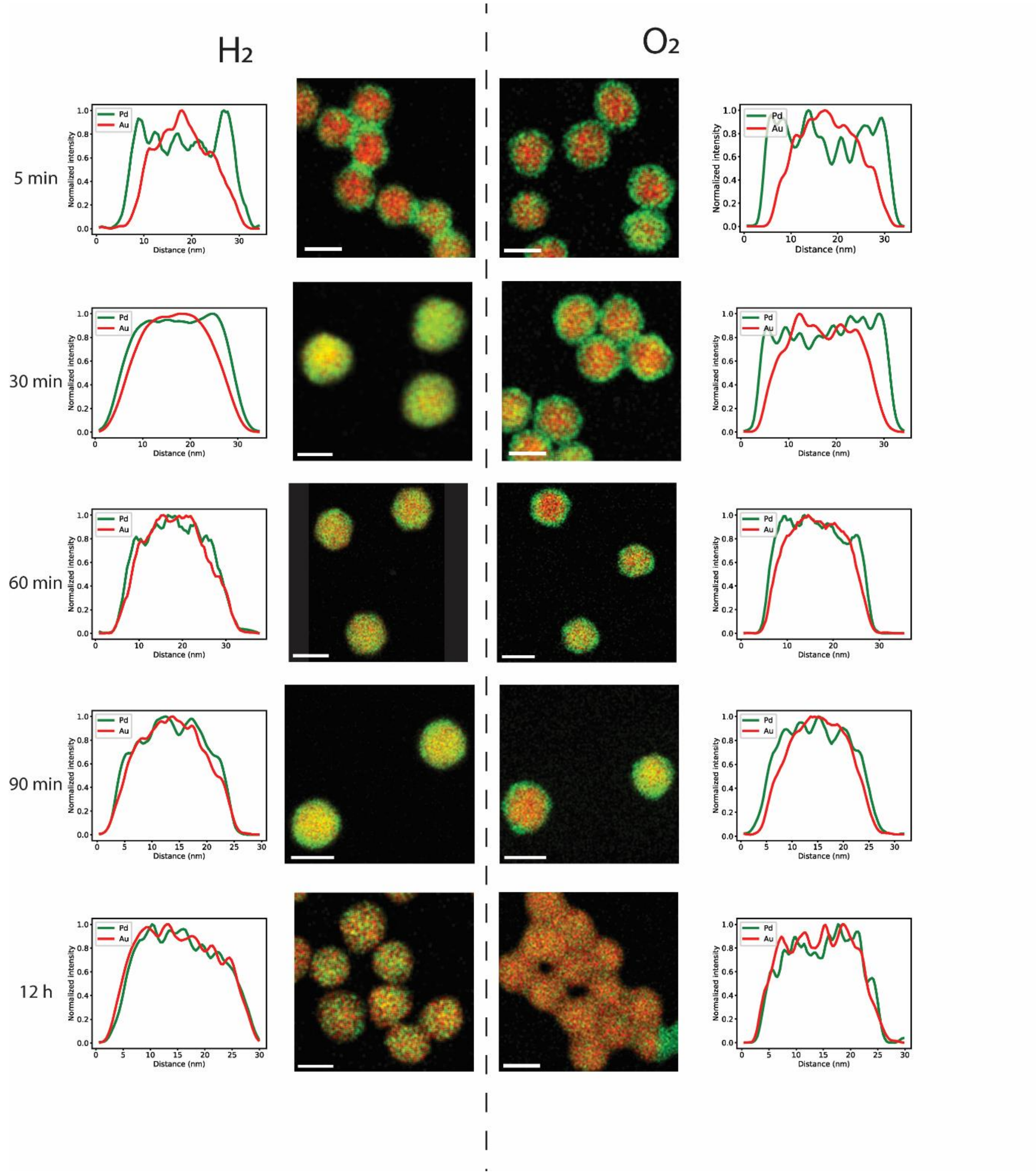

Figure S3. ***Ex situ*** **TEM measurements on the alloying of Au-core Pd-shell NPs in reducing and oxidizing gas atmospheres.** EDX maps and corresponding line scans after several min at 375 °C under H_2 and O_2 atmosphere. This data was used to compose Figure 2c.

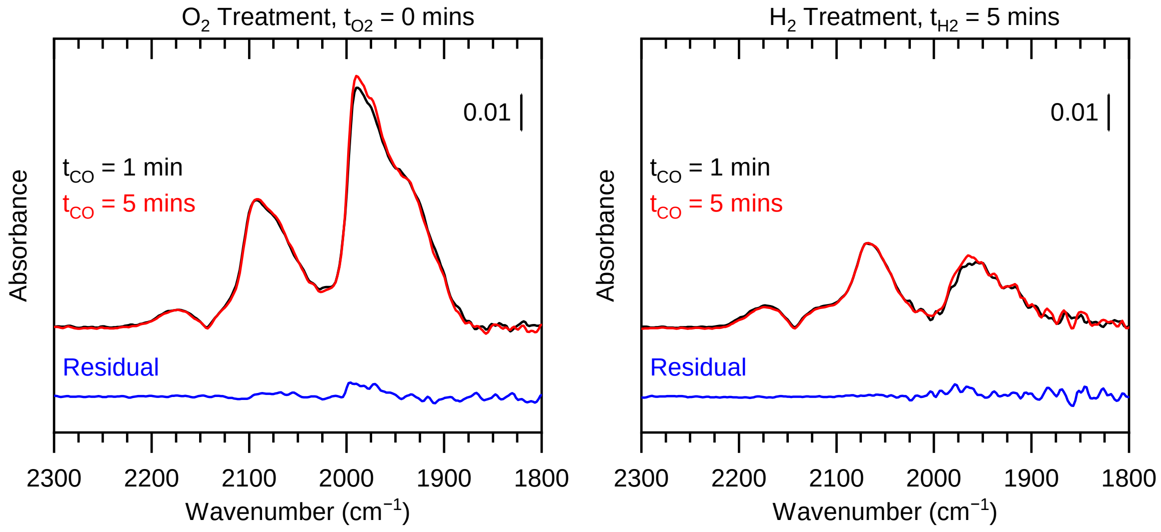

Figure S4. Representative time-dependent DRIFT spectra demonstrates a negligible impact on CO absorbance between (black) 1 and (red) 5 mins in the CO gas environment by the (blue) differential spectra for (a) an O_2 treated and (b) H_2 treated sample in Figure 3. The exchange time for the gas environment of the low-volume DRIFTS cell was measured to be <10 s previously.[1]

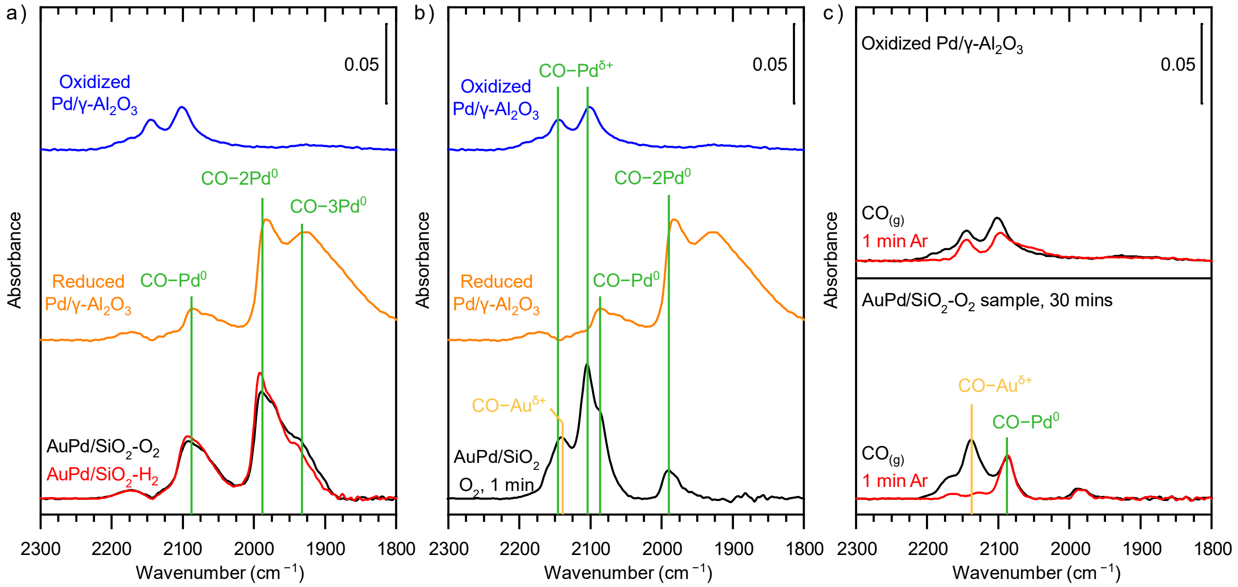

Figure S5. Spectroscopic evidence for DRIFTS ν(C−O) peak assignments for CO adsorption on core-shell AuPd/SiO$_2$ presented in Figure 3. (a) The vibrational frequencies for the peaks assigned to three-fold hollow (CO−3Pd^0), bridge (CO−2Pd^0) and linearly bound (CO−Pd^0) CO to metallic Pd for the H_2 pretreated AuPd/SiO$_2$ samples are in excellent agreement with those of H_2 pretreated 5nm Pd/γ-Al$_2$O$_3$. (b) The vibrational frequency for the peak assigned to linearly bound CO to cationic Pd (CO−Pd$^{\delta+}$) for the O_2 treated AuPd/SiO$_2$ sample is in excellent agreement with those of O_2 treated 5nm Pd/γ-Al$_2$O$_3$. However, the vibrational frequency for the peak assigned to linearly bound CO to cationic Au (CO−Au$^{\delta+}$)

could also be attributed to linear bound CO to cationic Pd (CO−$Pd^{δ+}$). (c) The assignment of linearly bound CO to cationic Au (CO−$Au^{δ+}$) is confirmed by rapid desorption indicative of Au when switching from a CO environment to a pure Ar environment for the AuPd/SiO_2 sample compared to Pd/γ-Al_2O_3. Each panel denotes a 0.05 absorbance intensity legend. H_2 pretreatment: 5% H_2/Ar, 250 °C, 45 min; O_2 treatment: (10% O_2/Ar, 375 °C, 1 min); CO adsorption conditions: 0.1 % CO/Ar, 25 °C.

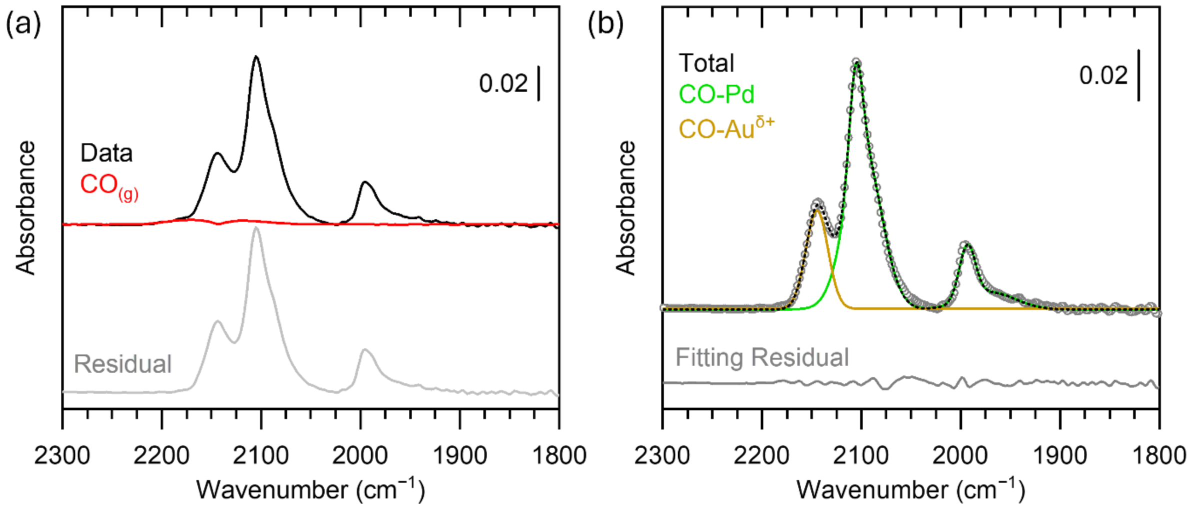

Figure S6. Representative DRIFT spectra for quantification of CO-Pd related peaks by sequential (a) gas phase CO removal and (b) peak fitting with Gaussians to distinguish CO-$Au^{δ+}$ from CO-Pd related peaks as illustrated by the spectrum after a 1 min O_2 treatment in Figure 3. The Gaussian fitting procedure was not necessary for quantification of the H_2 treatment spectra because there were no notable spectral features associated with CO-Au.

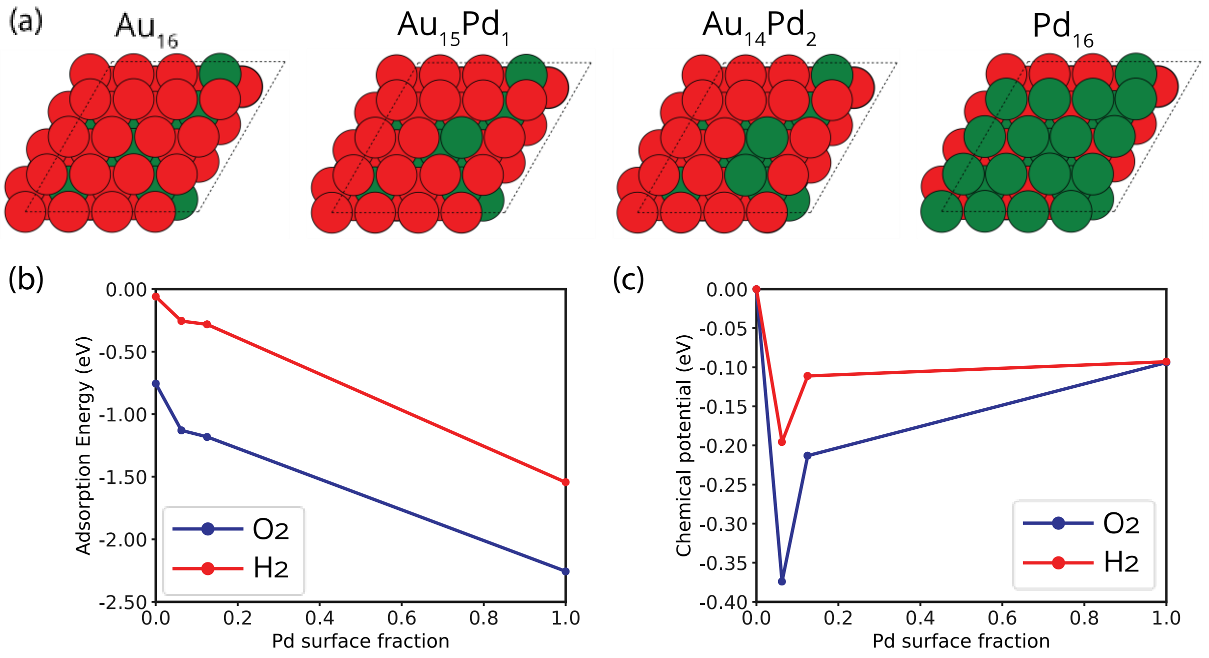

Figure S7. DFT calculations indicate a stronger driving force for Pd-surface enrichment in O_2. (a) Four structure models showing the top views of calculated (111) surfaces. From left to right: Au_{16}@PdAu, Pd_{16}@PdAu, $Au_{15}Pd_1$@PdAu, and $Au_{14}Pd_2$@PdAu (Pd/green, Au/red). The subsurface layers consisted of 36 atm% Pd randomly distributed in Au. (b) DFT-calculated adsorption energy of H_2 (red) and O_2 (blue) on Au-Pd (111) surface. Pd termination is favored for both adsorbates, with the lowest adsorption energy achieved for O_2. (c) DFT-calculated chemical potential on Au-Pd (111) surfaces upon H_2 or O_2 adsorption. The chemical potential is minimized for O_2 adsorption the $Au_{15}Pd_1$ surface.

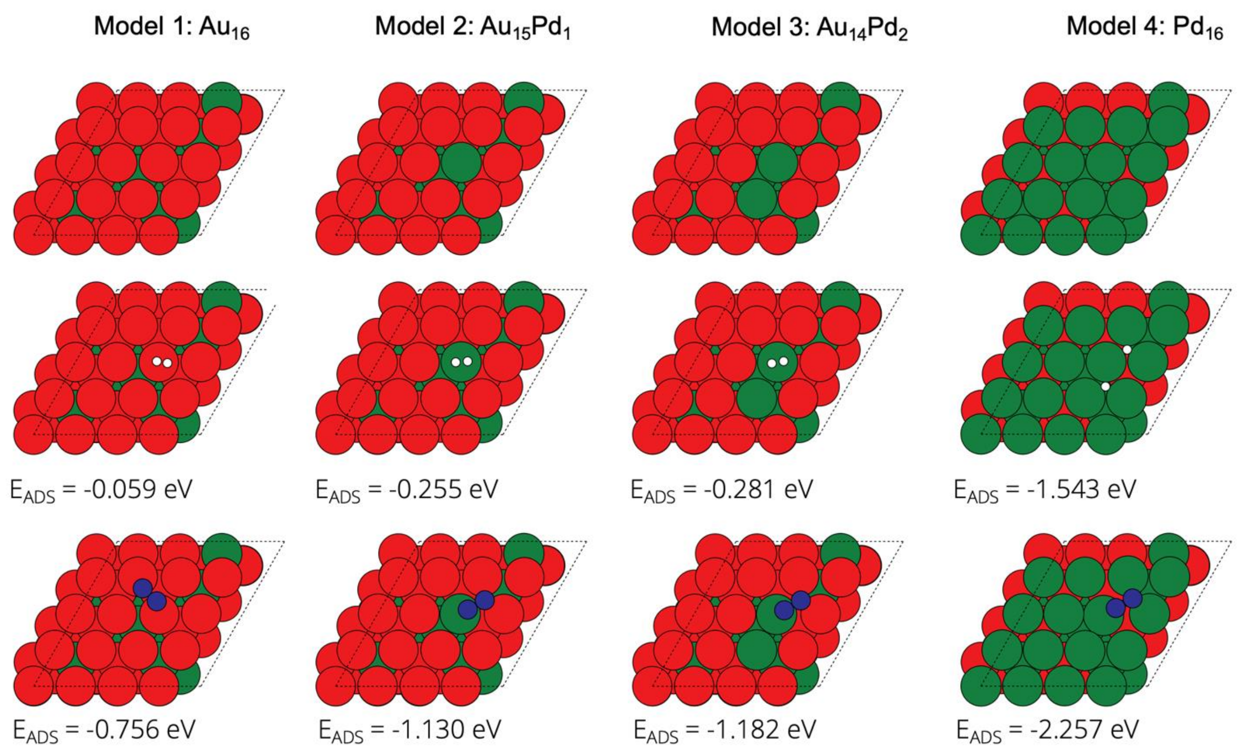

Figure S8. DFT-calculated adsorption energy (eV) of H_2 (white) and O_2 (blue) on Au-Pd (111) surface. Four DFT structure models were considered: top views of calculated (111) surfaces - Au_{16}@PdAu, Pd_{16}@PdAu, $Au_{15}Pd_1$@PdAu, and $Au_{14}Pd_2$@PdAu (Pd/green, Au/red), with Hydrogen/white and Oxygen/blue. The data from DFT calculations can be accessed on the GitHub repository at https://github.com/atomisticnet/2024-AuPd-DFT-data.[2]

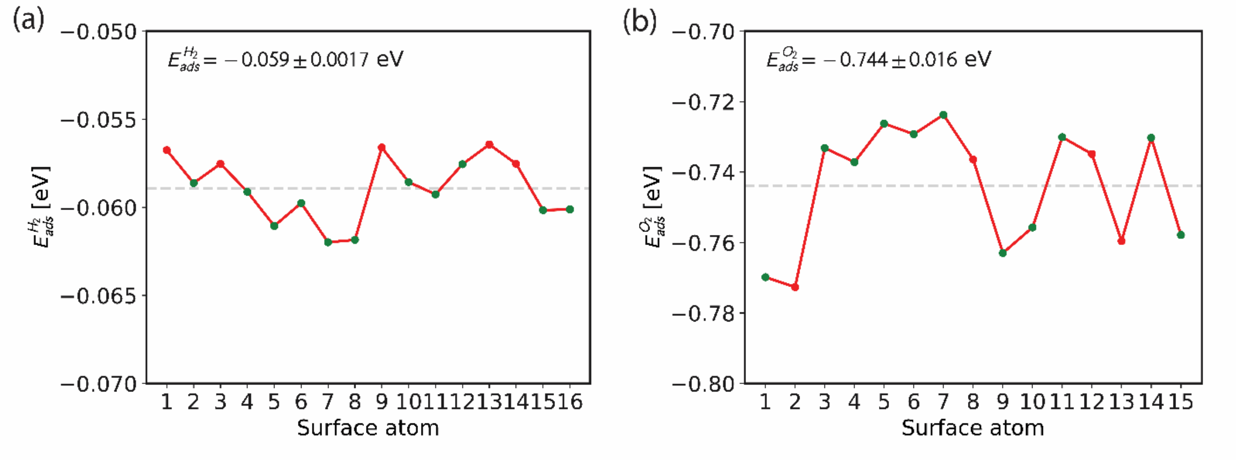

Figure S9. Effect of subsurface Pd on the DFT-calculated adsorption energies of (a) H_2 and (b) O_2 on the Au_{16} surface. The red dots represent surface atoms with no Pd atoms in the subsurface, and the green dots represent surface atoms with at least one Pd atom in the subsurface.

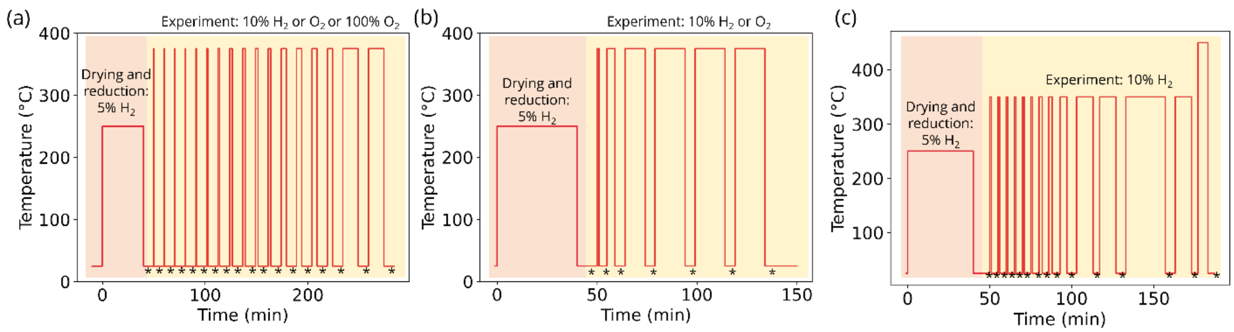

Figure S10. Temperature and gas profile of the *in situ* gas phase TEM experiments. The asterisks (*) represent the points where the beam was on and images were taken. The red shadowed region corresponds to the drying and reduction step, of 45 min at 250 °C in a reducing atmosphere (5% H_2 in Ar). The yellow shadowed region corresponds to the experiment itself, with 10% H_2 or O_2. All gases are at atmospheric pressure and balanced with Ar. (a) Experiment at 375 °C with 18 data points at time 0.0, 0.25, 0.5, 0.75, 1.0, 1.5, 2.5, 3.5, 5.0, 7.5, 10.0, 12.5, 15.0, 20.0, 25.0, 30.0, 45.0 and 60.0 minutes and a total dose of 15570 e^-/A^2. (b) Experiment at 375 °C with less data points, at time 0.0, 1.0, 5.0, 15.0, 30.0, 45.0 and 60.0 minutes and a total dose of 6055 e^-/A^2. (c) Experiment at 350 °C with 14 data points at time 0.0, 1.0, 2.0, 3.0, 4.0, 5.0, 6.0, 8.0, 10.0, 15.0, 25.0, 35.0, 50.0 and 60.0 minutes and a total dose of 7260 e^-/A^2.

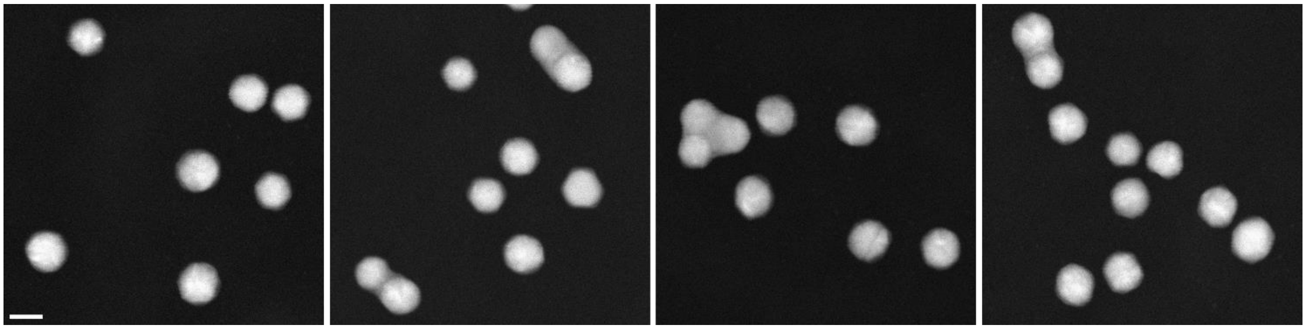

Figure S11. Regions imaged under H_2 at t = 0 min. These NPs were followed throughout the entire experiment. Scale bar is 20 nm.

Data analysis procedure: from HAADF-STEM images to measuring alloying

1. Selecting the regions of a histogram

The datasets to analyze consisted of a stack of 18 HAADF-STEM images corresponding to different time points, where the sample was cooled down to room temperature to image. The detailed acquisition parameters are found in the methodology section. The image at t = 0, the starting point, shows the Au-Pd core-shell NPs (Figure S9a). Three different gray value distributions are present in the image: the black values for the background, the white values for the Au-core and the grey values for the Pd-shell. Those are marked as squares in Figure S9a, and the respective histograms are shown in Figure S9b. In a histogram of a full NP, these three distributions are combined, and the Pd-shell grey values show as a shoulder between the black values of the background and white values of the Au-core (Figure S9c).

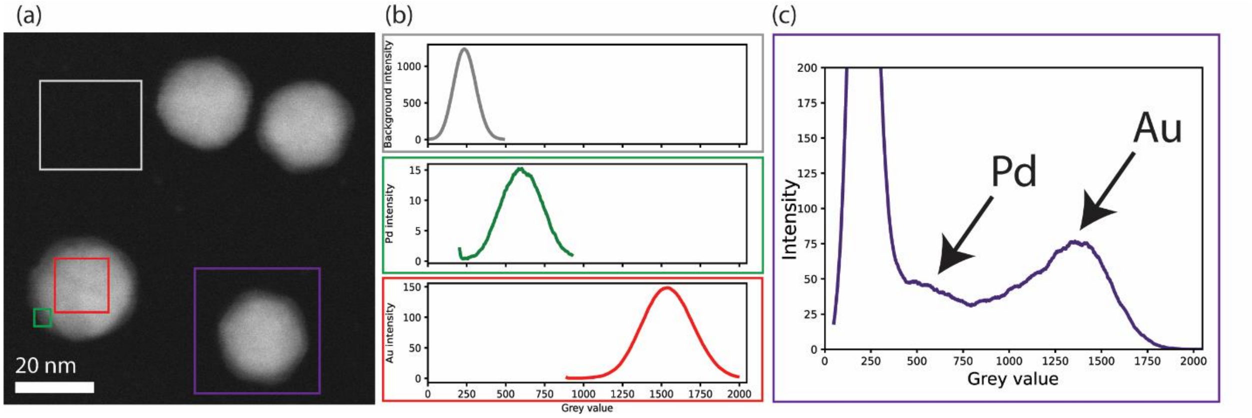

Figure S12: Grey value histograms of different regions on an image. (a) example HAADF-STEM image at t = 0 with different regions corresponding to different gray values. (b) grey values corresponding to the different colored squares marked on the image. From top to bottom: background, Pd shell and Au core. (c) histogram of a NP at t = 0 (combination of the three components).

To determine which grey values belong to the Pd-shell among the images, one histogram of the Pd-shell per NP was obtained (similar regions to the green square in Figure S9a, which histogram was like the green square in Figure S9b). For each histogram, the mean grey value and standard deviation were collected. This mean and standard deviation were averaged among all NPs, and the result corresponds to the average Pd-shell grey value at time = 0 min and its standard deviation. This is the region of interest in the histograms, that we will follow over time. However, taking the full standard deviation, we noticed that the Pd-shell grey values on the histogram overlapped with the background peak. That is why we decided to take the mean grey value ± 0.5 the standard deviation of the average histogram of the Pd-shell values as the region of interest for our further analysis.

2. Histogram evolution upon heating

During the alloying process of 1 h at 375 °C, the heating was interrupted at several time points, and the peak corresponding to the Pd shell slowly disappeared. An example dataset of a NP is shown in Figure S10: an image of the NP at every time point (Figure S10a) and the evolution of the grey value histograms (Figure S10b). Over time, the shell stops being visible in the HAADF-STEM images (Figure S10a) and the grey shoulder related to the Pd shell also disappears (Figure S10b). Note that the Pd-shell region of interest is in marked in yellow in the histograms of Figure S10b. Similar stacks of images and their histograms have been acquired for a total of 29 NPs in the H_2 experiment and 31 NPs in the O_2 experiment. While we know from EDX that the final state of the NPs is a full alloy (see results section, Figure S15 and S16), the histogram at t = 60 min still shows intensity at the Pd-shell related grey values. This probably originates from the thickness contrast also present in the images.

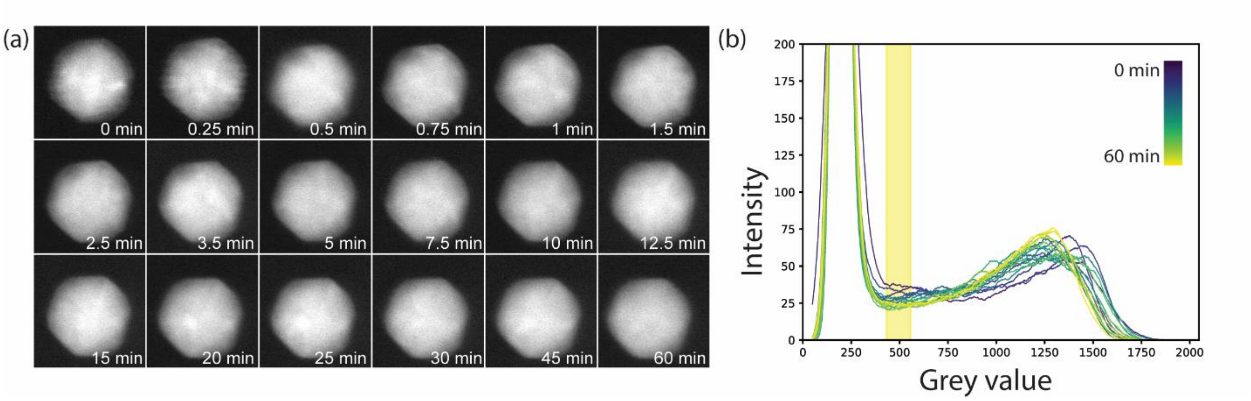

Figure S13. (a) Example dataset of one NP and (b) its gray value histogram over time. The shadowed area corresponds to the Pd-shell area used for analysis.

3. Analysis method: monitoring the alloying process by the disappearance of the Pd shell peak

To monitor the alloying progress at each time step, the area between the histogram at a certain time point (i) and the histogram at t = 60 min was computed. Figure S11 shows, for the histograms of a NP at times 0, 1 and 15 min, how the area between the histograms is measured. This area calculation was performed individually for each NP at each time point, and it allows deconvoluting the elemental and thickness contrast, thereby ensuring that only the z-contrast of Pd is used for the calculation of alloying dynamics.

The Pd-shell related areas varied among NPs, because of their size or shell thickness. To study the alloying behavior of all NPs together, the area values were normalized by dividing them by the area between t = 0 and t = 60, which corresponds to the Pd-shell area of the core-shell NP (Equation 1). Therefore, the core-shell NPs present at time = 0 min have a normalized Pd-shell area of 1, while the alloyed NPs at t = 60 min have a normalized Pd-shell

area of 0 (note that here t = t_{60}, and that calculating the area between the same curve is always 0).

(Equation 1)
$$Normalized\ area = \frac{Area\ between\ t_i\ and\ t_{60}}{Area\ between\ t_0\ and\ t_{60}}$$

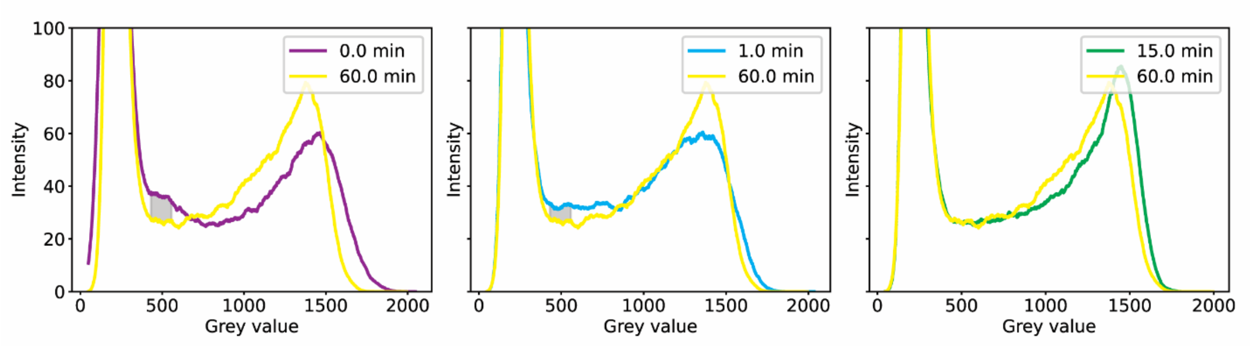

Figure S14. Evolution of the area corresponding to the Pd-shell during the heating process. In yellow, the histogram of the alloyed NP at time = 60 minutes. In purple, blue and green, three histograms at 0 min (a), 1 min (b) and 15 min (c), with the area between them in the Pd-shell region marked in grey.

4. Dataset cleaning

For some specific particles, the core-shell structure was not well-visible at time = 0. For these NPs, the Pd-shell related area at time 0 was very small, as well were the differences between time points. Therefore, we decided to remove these NPs from analysis, by setting a threshold of the Pd-shell area at t = 0. If this area was smaller than 500 units, the dataset for the NP was not used. In this manner, 1NP was removed from the H_2 experiment, 4 NPs were removed from the O_2 experiments and 5 NPs were removed from the experiment at 350 °C and 18 NPs for the 100% O_2 experiment. The larger amount of particles removed from the experiment at 100% O_2 originates from slightly poorer image quality at time = 0 min in two out of the four regions that were imaged, probably because of a slightly higher vacuum level in the microscope, leaving us with 14 NPs for the data analysis.

The normalized Pd-shell area should by definition oscillate between 1 (core-shell) and 0 (alloy). However, this was not the case for all NPs at all time points, since there is also diffraction and thickness contrast present in the image, that alter the shape of the grey value histograms. Since we use an average to get the overall behavior of all NPs (see next step), the extreme values were excluded, so that the normalized areas could only be [-0.5, 1.5]. If extreme values were found for more than half of the histograms, the whole dataset was removed. This only happened once in the 10% O_2 experiment.

5. Including all NPs in the analysis

Figure S12a shows all the data points for the normalized Pd-shell area in all the NPs followed through the H_2 experiment. After calculating the normalized Pd-shell area of each particle at each time point, it was possible to average it among particles, since all the initial and final states were the same (core-shell and alloyed, respectively). The result of averaging each data point is shown at Figure S12b. When doing so, we noticed that while there is quite a big variation among particles, there is a general trend. This provides the information on the alloying dynamics. In this case, in Figure S12b (part of Figure 5d in the main text) shows that the alloying process is completed after 10 minutes, since the values decrease from 1 to 0 in this time range.

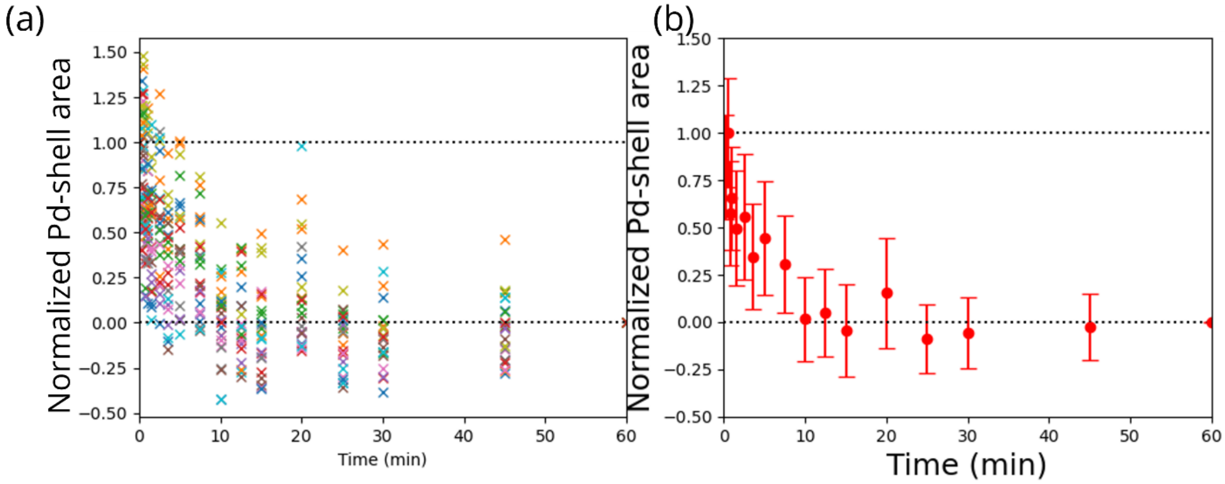

Figure S15. Normalized area evolution over time at 375 °C. (a) variation of normalized area between NPs at selected time points. (b) normalized Pd-shell area evolution over time averaged from 24 NPs in the H_2 experiment.

6. Validating the methodology

To validate the methodology described above, we performed an *in situ* heating experiment under vacuum, increasing the temperature from 25 °C to 600 °C and taking an EDX map at each temperature. The EDX data was analyze with a published methodology,[3] and the result is shown in Figure S13a. Figure S13b shows the analysis of the HAADF images of the same temperature steps analyzed by the method described in this work. The two results are very similar, validating our approach of quantifying the alloying degree by monitoring the decrease in Pd-shell related area in the grey-value histograms.

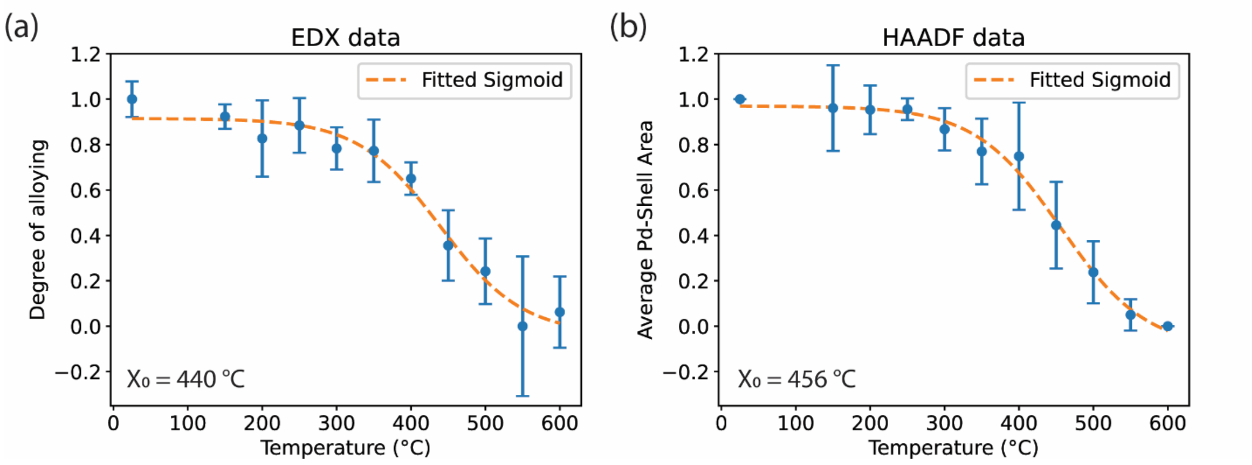

Figure S16. Degree of alloying calculated from *in situ* heating under vacuum experiments. (a) from EDX mapping. (b) from the approach used in the *in situ* gas cell experiments described above. X_0 is the midpoint of the sigmoid fit.

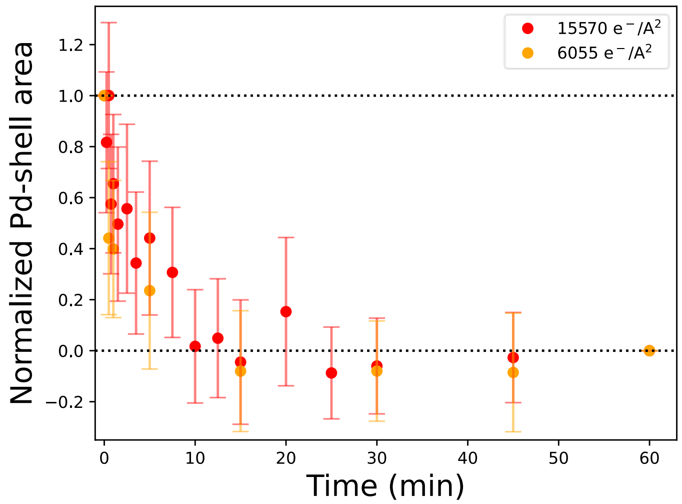

Figure S17. The NPs irradiated with less than half the electron dose show the same alloying dynamics than the NPs in the H_2 experiment. Comparison of normalized Pd-shell area in regions that were irradiated with more (15570 e^-/A^2) or less (6055 e^-/A^2) electron dose.

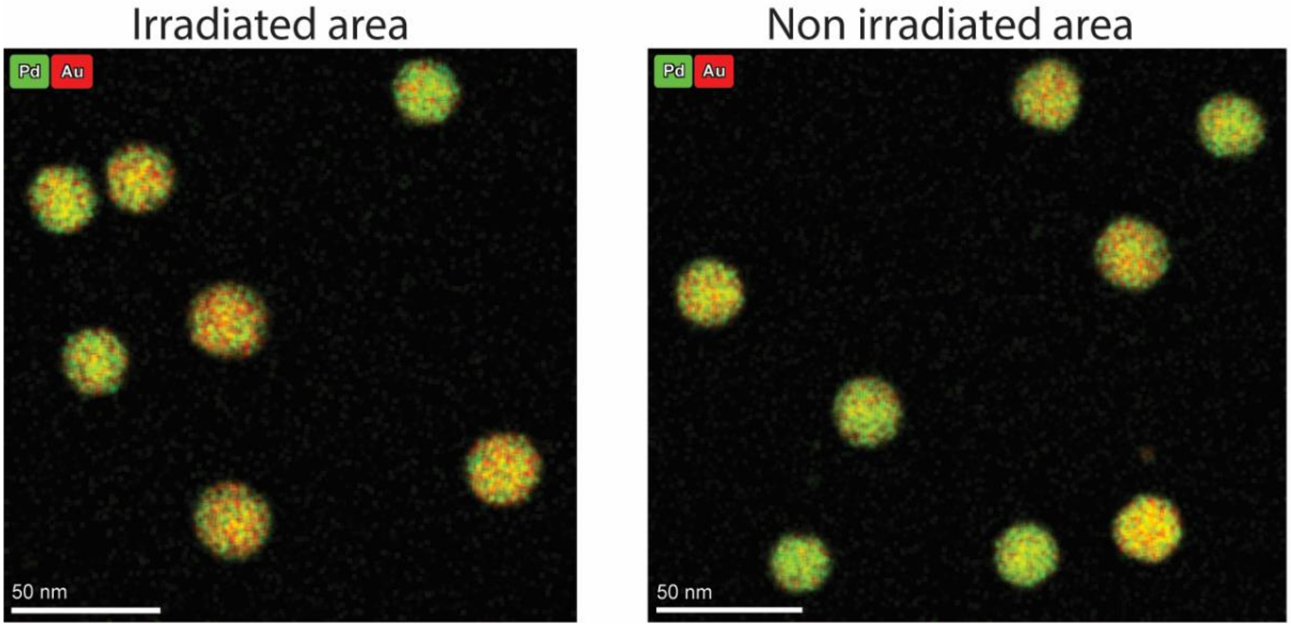

Figure S18. EDX mapping after the *in situ* H_2 STEM experiment at 375 °C. (a) irradiated area. (b) non irradiated area.

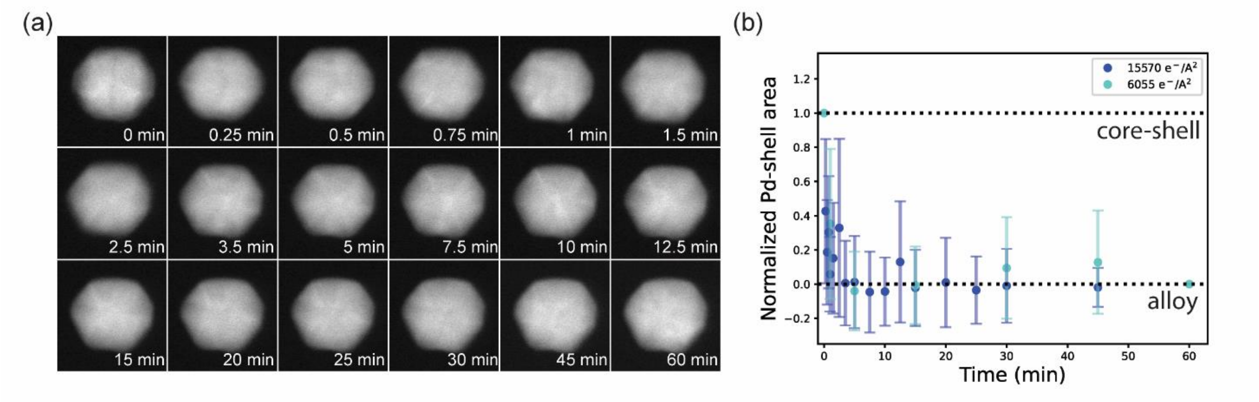

Figure S19. In situ alloying of Au-Pd core-shell NPs under 1 atm 10% O_2 in Ar (a) HAADF-STEM images of an example NP heated over 60 minutes at 375 °C. (b) Decrease of Pd-shell area over time normalized over 31 nanoparticles, in regions that were irradiated with more (15570 e^-/A^2) or less (6055 e^-/A^2) electron dose.

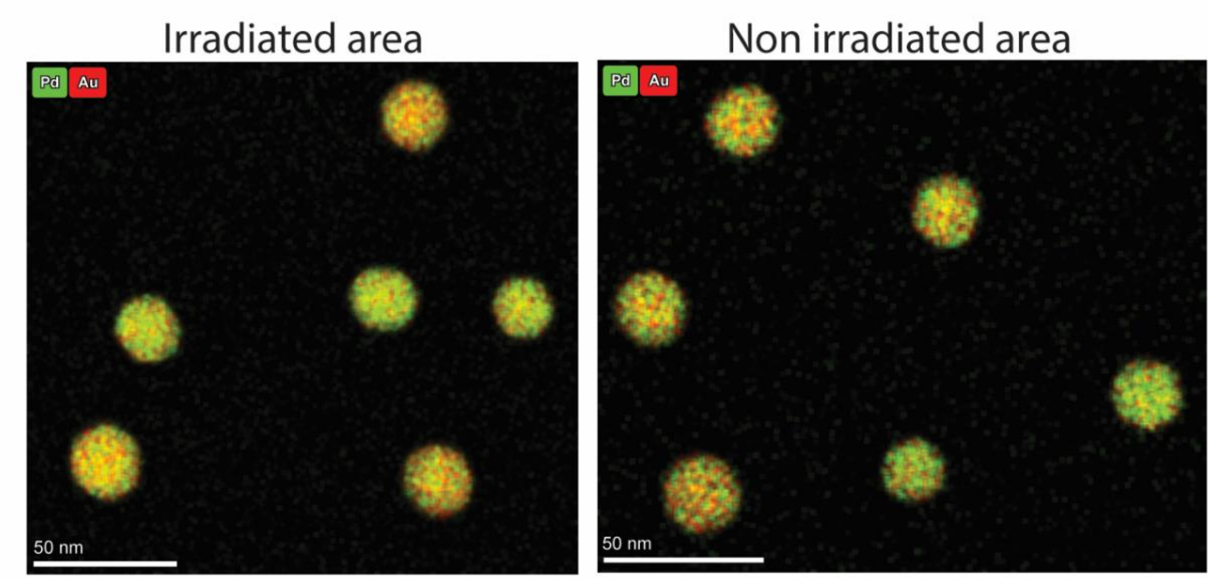

Figure S20. EDX mapping after the *in situ* O_2 STEM experiment at 375 °C. (a) irradiated area. (b) non irradiated area

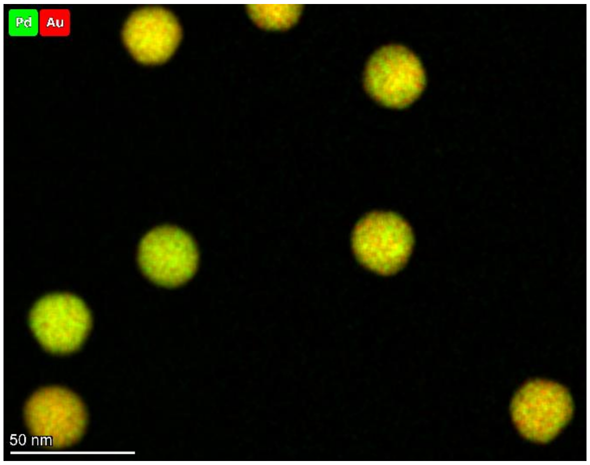

Figure S21. EDX mapping after an experiment of 1 h at 375°C under O_2 without beam irradiation at all.

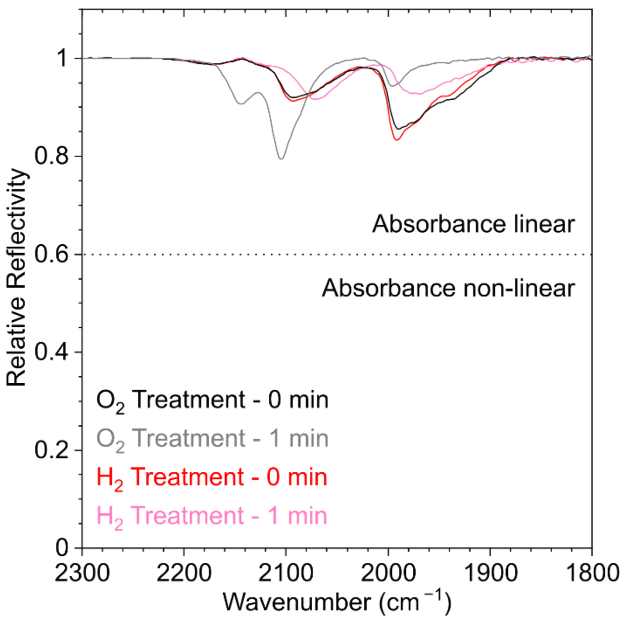

Figure S22. DRIFT spectra in relative reflectivity units for CO adsorption on $AuPd/SiO_2$ presented in Figure S4. The relative reflectivity for all DRIFTS spectra is above 0.75 which indicates that absorbance units are expected to be nearly proportional to adsorbate concentration.[4]

Table S1. Vibrational frequency of the peak maxima and species assignment for ν(C−O) features.

Notation	Assignment	Frequency (cm^{-1})
$CO_{(g)}$	Gas phase CO	2173, 2120
$CO{-}Au^{\delta+}$	Linear CO on cationic Au	2143
$CO{-}Pd^{\delta+}$	Linear CO on cationic Pd	2105
$CO{-}Pd^{0}$	Linear CO on metallic Pd	2088
$CO{-}2Pd^{0}$	Bridge CO on metallic Pd	1985
$CO{-}3Pd^{0}$	Three-fold hollow CO on metallic Pd	1935

References:

(1) Connor, C. R. O.; Le, S.; Kim, T.; Reece, C. Resolving Transient Responses of Catalyst Systems to Reactant Stream Modulation Using Low Volume HVC DRIFTS Reactor. No. 21176.

(2) Draijer, K.; Artrith, N. *Au-Pd DFT Data*. https://github.com/atomisticnet/2024-AuPd-DFT-data (accessed 2024-02-01).

(3) van der Hoeven, J. E. S. S.; Welling, T. A. J. J.; Silva, T. A. G. G.; Van Den Reijen, J. E.; La Fontaine, C.; Carrier, X.; Louis, C.; van Blaaderen, A.; de Jongh, P. E. In Situ Observation of Atomic Redistribution in Alloying Gold–Silver Nanorods. *ACS Nano* **2018**, *12* (8), 8467–8476. https://doi.org/10.1021/acsnano.8b03978.

(4) Sirita, J.; Phanichphant, S.; Meunier, F. C. Quantitative Analysis of Adsorbate Concentrations by Diffuse Reflectance FT-IR. *Anal. Chem.* **2007**, *79* (10), 3912–3918. https://doi.org/10.1021/ac0702802.
